# The Latent Dirichlet Allocation model with covariates (LDAcov): A case study on the effect of fire on species composition in Amazonian forests

**DOI:** 10.1002/ece3.7626

**Published:** 2021-05-05

**Authors:** Denis Valle, Gilson Shimizu, Rafael Izbicki, Leandro Maracahipes, Divino Vicente Silverio, Lucas N. Paolucci, Yusuf Jameel, Paulo Brando

**Affiliations:** ^1^ School of Forest, Fisheries, and Geomatics Sciences University of Florida Gainesville Florida USA; ^2^ Department of Statistics Federal University of Sao Carlos Sao Paulo Brazil; ^3^ Instituto de Pesquisa Ambiental da Amazonia Brasilia Brazil; ^4^ Departamento de Biologia Universidade Federal Rural da Amazônia Capitão Poço Brazil; ^5^ Departamento de Biologia Geral Universidade Federal de Viçosa Viçosa Brazil; ^6^ Department of Earth System Science University of California, Irvine Irvine California USA

**Keywords:** Amazon, biodiversity, community ecology, forest fire, forest fragmentation, mixed‐membership model, multivariate statistics

## Abstract

Understanding and predicting the effect of global change phenomena on biodiversity is challenging given that biodiversity data are highly multivariate, containing information from tens to hundreds of species in any given location and time. The Latent Dirichlet Allocation (LDA) model has been recently proposed to decompose biodiversity data into latent communities. While LDA is a very useful exploratory tool and overcomes several limitations of earlier methods, it has limited inferential and predictive skill given that covariates cannot be included in the model. We introduce a modified LDA model (called LDAcov) which allows the incorporation of covariates, enabling inference on the drivers of change of latent communities, spatial interpolation of results, and prediction based on future environmental change scenarios. We show with simulated data that our approach to fitting LDAcov is able to estimate well the number of groups and all model parameters. We illustrate LDAcov using data from two experimental studies on the long‐term effects of fire on southeastern Amazonian forests in Brazil. Our results reveal that repeated fires can have a strong impact on plant assemblages, particularly if fuel is allowed to build up between consecutive fires. The effect of fire is exacerbated as distance to the edge of the forest decreases, with small‐sized species and species with thin bark being impacted the most. These results highlight the compounding impacts of multiple fire events and fragmentation, a scenario commonly found across the southern edge of Amazon. We believe that LDAcov will be of wide interest to scientists studying the effect of global change phenomena on biodiversity using high‐dimensional datasets. Thus, we developed the R package LDAcov to enable the straightforward use of this model.

## Graphical abstract

We introduce a modified Latent Dirichlet Allocation model (called LDAcov) which allows for the incorporation of covariates, enabling statistical inference on the drivers of change of latent communities, spatial interpolation of results, and prediction based on future environmental change scenarios. We illustrate LDAcov using data from two experimental studies on the long‐term effects of fire on southeastern Amazonian forests in Brazil. We believe that LDAcov will be of wide interest to scientists studying the effect of global change phenomena on biodiversity using high‐dimensional datasets.

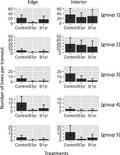

## 1 INTRODUCTION

Understanding and predicting how species composition has been and will be altered by global change phenomena is key to sustaining biodiversity and ecosystem functioning. However, biodiversity data are highly multivariate, containing information on tens to hundreds of species in a given location and time (Ramette, 2007; Warton et al., 2015). Thus, dimension reduction techniques are required to generate interpretable findings from these data (McCune et al., 2002). Clustering and ordination have been the main approaches in ecology to reduce the dimensionality of biodiversity data (Legendre & Legendre, 2012). Clustering approaches have been extensively used in Ecology since at least the 1920s (Legendre & Legendre, 2012). Although hard‐clustering approaches have dominated the field, few ecological theories predict the sharp delineations implied by these methods (Legendre & Legendre, 2012). Importantly, these hard‐clustering methods will assign a given location to a single group, limiting the ability of these approaches in detecting gradual changes in species composition across environmental gradients.

Differently from cluster analysis, ordination is typically the method of choice to identify general gradients in highly multivariate data (Legendre & Legendre, 2012). Unconstrained ordination methods (e.g., principal component analysis [PCA], correspondence analysis [CA], principal coordinate analysis [PCoA], and nonmetric multidimensional scaling [NMDS]) enable the visualization of the variability in multivariate data in a space with reduced dimensionality (typically two; Hui et al., 2015), whereas constrained ordination methods (e.g., redundancy analysis [RDA] and canonical correspondence analysis [CCA]) allow for statistical testing of environment–species composition associations (Legendre & Legendre, 2012; Ramette, 2007). The main limitations associated with these methods are the poor interpretability of their results and lack of ability to make predictions. Because all the information from ecological data is typically condensed into a square dissimilarity matrix prior to the analysis, it is challenging to determine how individual species contribute to the final results, hampering the ability to interpret how the different axis scores relate to the observed species composition at each site. Finally, most cluster and ordination methods used for biodiversity research are algorithm‐based techniques with no underlying statistical model. As a result, few of these methods can be used to make predictions and there is often no quantification of uncertainty associated with their results, a critical limitation for inference and prediction purposes (Hui et al., 2015).

The Latent Dirichlet Allocation (LDA) model is a type of Bayesian mixed‐membership model that allows for realistic representation of both gradual and sharp changes in species compositions along environmental gradients (Valle et al., 2014). Instead of representing biogeographical regions with sharp boundaries, LDA can represent biome transition zones and ecotones as mixed‐membership areas. The ability of LDA to represent the blending of assemblages in these transition zones has been shown repeatedly in previous articles (Valle et al., 2014, 2018). Importantly, LDA estimates the proportion of each group in each sampling unit, a much more straightforward quantity to interpret than results from ordination methods (e.g., PCA or NMDS scores). LDA models have become increasingly popular, being used to model spatial and temporal change in communities for a wide range of taxa across a diverse set of systems (Christensen et al., 2018; Dietzel et al., 2019; Knott et al., 2019; Muhlfeld et al., 2020; Sommeria‐Klein et al., 2019; Valle et al., 2014, 2018). Unfortunately, despite its usefulness for exploratory analysis, LDA is limited in its ability to make inference and predictions given that covariates are not included in the model.

The goal of this article is to introduce a modified LDA model that allows for inference and prediction on the abundance of individual groups. We first describe the model and then, using simulated data, we show that the model can retrieve well the true parameter values. Finally, we apply the developed model to two experimental studies on the long‐term effects of fire on southeastern Amazonian forests in Brazil. These forests are located in the driest portion of the biome and are known to be relatively resistant to a single fire, but are dramatically impacted by repeated fires, especially under extreme climatic conditions (Balch et al., 2015). Several studies have shown that fires cause high tree mortality and significantly impact forest structure, diversity, and function (Balch et al., 2011, 2015; Brando et al., 2014, 2016; Brando, Silverio, et al., 2019; Nobrega et al., 2019). Furthermore, windstorms and drought often exacerbate fire and its effects on forests (Brando et al., 2014; Silvério et al., 2019). Acting synergistically, these processes induce changes that may ultimately lead to the “savannization” of parts of the Amazon (i.e., a collapse of tropical rainforests, transforming them into a low‐biomass savanna‐like biome) (Nobre et al., 2016). Unfortunately, current understanding of the impact of fire on species composition is still limited, a gap that this study aims to help fill.

## 2 METHODS

### 2.1 Model description

The LDA model with covariates (LDAcov) embeds a negative binomial regression within LDA to determine how the number of individuals in each group is influenced by covariates. Let $n_{\mathrm{lsk}}$ be the number of individuals in location *l* and group *k* from species *s*. We assume that the number of individuals in location *l* assigned to group *k* ($n_{l.k}$) across all species (i.e., $n_{l.k}=\sum_{s=1}^{S}n_{\mathrm{lsk}}$) is given by a negative binomial regression:

$$n_{l.k}∼N\text{Binom}\left(\exp\left(x_{l}^{T}\beta_{k}\right),N\right).$$

where $E\left[n_{l.k}\right]=\exp\left(x_{l}^{T}\beta_{k}\right)$ and *N* is a parameter that captures over‐dispersion. Furthermore, $\beta_{k}$ is a vector of group‐specific regression parameters and $x_{l}^{T}$ is the location‐specific design vector containing a leading 1 (for the intercept) and the covariates for location *l*. Next, we assume that:

$$\left[n_{l1k},\ldots ,n_{\mathrm{lSk}}\right]∼\text{Multin}\left(n_{l.k},\phi_{k}\right)$$

In this expression, $\phi_{k}$ is a vector of group‐specific probabilities that sum to one. Each element $\phi_{\mathrm{ks}}$ within this vector describes the relative abundance of species *s* in group *k*, this way characterizing the species composition of this group. Notice that both $n_{\mathrm{lsk}}$ and $n_{l.k}$ are latent variables. The observations consist of the abundance of species *s* in location l ($n_{ls.}$) given by

$$n_{ls.}=\sum_{k=1}^{K}n_{\mathrm{lsk}}$$

We finish specifying our model by adopting the following prior distributions for $N,\phi_{k}$, and $\beta_{k}$:

N∼Unif(0,100)

$$\phi_{k}∼\text{Dirichlet}\left(\gamma 1\right)$$

$$\beta_{k}∼N\left(0,T\right)$$

where **T** is a diagonal matrix and 0<γ<1.

### 2.2 Gibbs sampler

Let $z_{\mathrm{il}}$ denote the group membership of individual *i* in location *l*, where $n_{\mathrm{lsk}}=\sum_{i=1}^{n_{l..}}I(z_{\mathrm{il}}=k,y_{\mathrm{il}}=s)$. To fit this model, we rely on a Gibbs sampler in which we iteratively sample each $\beta_{k}$, $\phi_{k}$, and $z_{\mathrm{il}}$. Below, we specify the full conditional distribution for each of these parameters.

The full conditional distribution for $\beta_{k}$ is given by:

$$p\left(\beta_{k}|\ldots \right)\propto \left[\prod_{l}N\text{Binom}(n_{l.k}|\exp\left(x_{l}^{T}\beta_{k}\right),N)\right]N\left(\beta_{k}|0,T\right)$$

To sample this vector of parameters, we rely on a slice‐sampler algorithm (Neal, 2003) applied sequentially to each element of this vector.

The full conditional distribution for *N* is given by:

$$p\left(N|\ldots \right)\propto \left[\prod_{k}\prod_{l}N\text{Binom}(n_{l.k}|\exp\left(x_{l}^{T}\beta_{k}\right),N)\right]\text{Unif}\left(N|0,100\right)$$

To sample this parameter, we also rely on a slice‐sampler.

Because of conditional conjugacy, the full conditional distribution for $\phi_{k}$ is a Dirichlet distribution, given by:

$$\begin{array}{c} p\left(\phi_{k}|\ldots \right)\propto \left[\prod_{l}\text{Multinom}\left(\left[n_{l1k},\ldots ,n_{\mathrm{lSk}}\right]|n_{l.k},\phi_{k}\right)\right]\text{Dirichlet}\left(\phi_{k}|\gamma \right)\\ =\text{Dirichlet}\left(\left[n_{.1k}+\gamma ,\ldots ,n_{.Sk}+\gamma \right]\right) \end{array}$$

where $n_{.sk}$ is the number of individuals from species s in group *k* across all locations (i.e., $n_{.sk}=\sum_{l}n_{\mathrm{lsk}}$).

Finally, as detailed in Appendix S1, conditional on $y_{\mathrm{il}}=s$, $z_{\mathrm{il}}$ is drawn from a categorical distribution with the following probability:

$$p\left(z_{\mathrm{il}}=k|y_{\mathrm{il}}=s,\ldots \right)=\frac{\frac{\left(n_{l.k}^{\left(‐i\right)}+N\right)}{\left(n_{\mathrm{lsk}}^{\left(‐i\right)}+1\right)}\phi_{\mathrm{ks}}\left(1‐p_{\mathrm{lk}}\right)}{\sum_{c=1}^{K}\frac{\left(n_{l.c}^{\left(‐i\right)}+N\right)}{\left(n_{\mathrm{lsc}}^{\left(‐i\right)}+1\right)}\phi_{\mathrm{cs}}\left(1‐p_{\mathrm{lc}}\right)}$$

where $n_{l.k}^{\left(‐i\right)}$ is the number of individuals in location *l* and group *k* after removing the *i*th individual. Similarly, $n_{\mathrm{lsk}}^{\left(‐i\right)}$ is the number of individuals in location *l*, group *k*, from species *s* after removing the *i*th individual. Finally, $p_{\mathrm{lk}}=N/\left(N+\exp\left(x_{l}^{T}\beta_{k}\right)\right)$.

### 2.3 Model fitting details

To aid the convergence of this model, it is critical for it to be initialized with sensible starting values. Furthermore, this model requires that the number of groups be a priori specified.

To obtain sensible starting values and to determine the optimal number of groups, we adopt a two‐stage approach. We first fit the data using an unconstrained LDA model (i.e., a model that does not include covariates and that does not have an embedded regression structure). This model identifies the optimal number of groups using a Bayesian nonparametric prior (i.e., the truncated stick‐breaking prior) and is described in detail in Albuquerque et al. (2019). Notice that, differently from an intercept‐only model, the unconstrained LDA model is very flexible because it estimates the proportion of each group at each location as separate parameters. Assuming the number of groups identified by the first model, we then use the $n_{\mathrm{lsk}}$ values provided by the unconstrained LDA model to initialize our model. We also initialize the regression coefficients $\beta_{k}$ by fitting a separate negative binomial regression for $n_{l.k}$ from each group.

**FIGURE 1:**
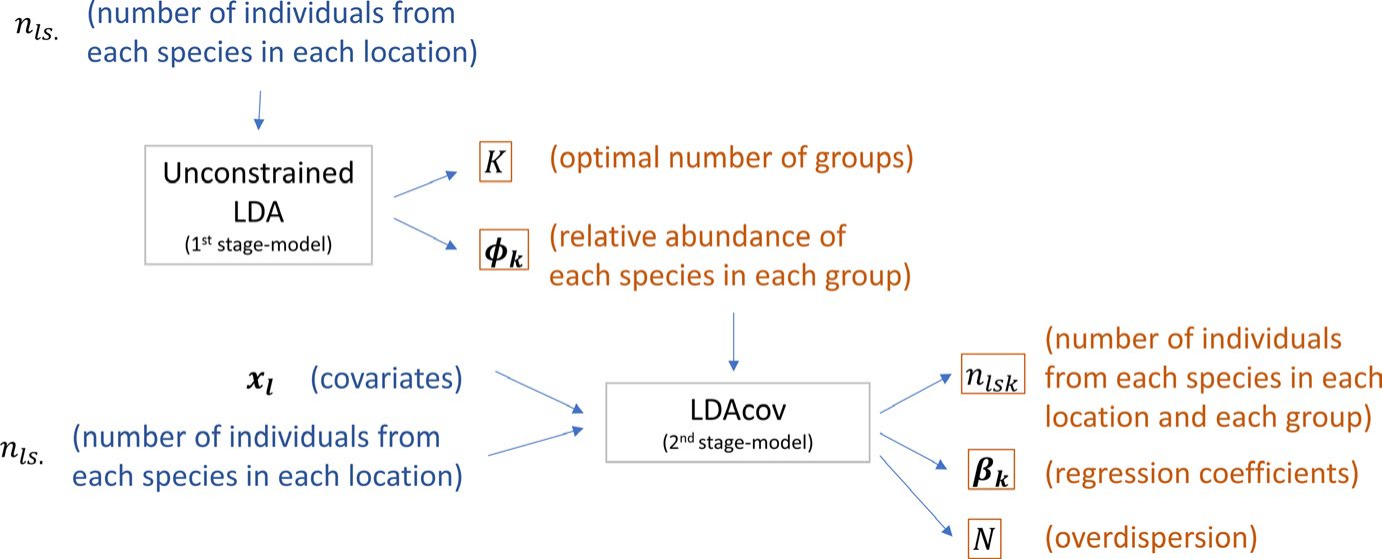
Illustration of the two‐stage approach to fitting LDAcov. First, an unconstrained LDA model is fit to abundance data $n_{\mathrm{ls}}.$ to estimate the optimal number of groups *K* and the species composition of each group $\phi_{k}$. Then, LDAcov is fitted using covariate information $x_{l}$ and abundance data $n_{\mathrm{ls}}$, yielding estimates of the regression coefficients $\beta_{k}$, the overdispersion parameter *N*, and the number of individuals in each species, location, and group $n_{\mathrm{lsk}}$. Descriptions for the data and parameters are displayed in blue and orange, respectively, and models have gray boxes

Differently from a standard regression in which the response variable is observed, fitting a regression model within an unsupervised method like LDA is challenging because the response variable is latent and has to be estimated together with the regression parameters. As a result, a misspecified regression model can negatively impact the (latent) response variable $n_{l.k}$, potentially mischaracterizing the identified communities. For this reason, we decided to use the posterior distribution of $\phi_{k}$ from the unconstrained LDA model as the posterior distribution from the LDAcov model. This way, even if none of the covariates are informative or if the model is misspecified, the communities identified by the unconstrained LDA model would still be the same as those identified by the LDAcov model. However, notice that, despite not estimating $\phi_{k}$, LDAcov still has to estimate $n_{\mathrm{lsk}}$ and all regression parameters. This two‐stage approach to fitting LDAcov is illustrated in Figure 1.

Our simulation results indicate that this two‐stage strategy is successful in retrieving the true values for $n_{\mathrm{lsk}}$ and $\beta_{k}$ and that using the posterior distribution of $\phi_{k}$ from the unconstrained LDA model consistently leads to better results than estimating $\phi_{k}$ within LDAcov (see Section 3). Nevertheless, our R package called LDAcov (available at https://github.com/gilsonshimizu/ldacov and described in detail in Appendix S2) enables the user to choose between estimating $\phi_{k}$ or relying on the posterior distribution of $\phi_{k}$ from the unconstrained LDA model.

### 2.4 Simulations

We simulate data to evaluate the ability of LDAcov to estimate the number of individuals in each group *k* and location *l* ($n_{l.k}$), the species composition ($\phi_{k}$), and the regression parameters $\beta_{k}$ of each group *k*. To illustrate how well the proposed method works in different settings, we varied the number of plots (set to 20, 40, 80, and 500) and the number of species (set to 80, 160, and 320), resulting in 12 scenarios. After removing rare species, the final number of species in these datasets was equal to 45, 65, and 92, respectively. To create the simulated datasets, we assumed that there were 3 groups and that each group was strongly influenced by just one out of the three covariates. To implement this assumption, the slope parameters for each group were equal to 2 for one covariate and 0 for the remaining covariates. Covariate values were simulated independently from a uniform distribution between −1 and 1.

### 2.5 Field data

We rely on datasets that arise from two experimental forest fires. Both experiments are located in a transitional forest in Mato Grosso, Brazil, in the southern part of the Amazon Basin (13°04′S, 52°23′W). In the first experiment, three 50 ha (50 × 1,000 m) plots bordering a crop field were established in 2004 (“Big‐plot” experiment from hereafter). In each plot, transects of 500 m in length and 20 m in width were created at 10, 30, 100, 250, 500, and 750 m from the forest edge and all trees with diameter at breast height (i.e., 1.3 m from the ground; dbh) greater than 20 cm were measured within these transects. One of these plots was left unburned (i.e., Control), one plot was burned thrice (2004, 2007, and 2010; hereafter “B3yr” treatment) and the remaining plot was burned yearly from 2004 to 2010, except in 2008 (hereafter “B1yr” treatment). Trees were measured in 2004, 2008, 2010, 2012, and 2016, always prior to the experimental fires. Additional details regarding this experiment are available in Balch et al. (2011).

The second experiment evaluated the effect of fuel addition and fire frequency on fire intensity and tree mortality. This experiment followed a randomized block design, with a total of 6 blocks and 4 plots of 40 m × 40 m within each block (“Block” experiment from hereafter). All trees with dbh greater than 5 cm were measured within these plots. Treatments consisted of unburned plots (control area), plots burned once in 2016 under natural conditions (i.e., no fuel addition), plots burned twice (2013 and 2016) under natural conditions, and plots burned twice (2013 and 2016) with fuel addition (50% increase in fine fuel loads) only before the 2013 fire. In this experiment, trees were measured yearly from 2011 to 2018, except for 2017, always prior to the experimental fires. Additional details regarding this experiment are available in Brando et al. (2016).

### 2.6 Data analysis for the fire experiments

For the “Big‐plot” experiment, we adopted the following regression structure for the number of individuals in each transect *l*, group *k,* and year *t* ($n_{l.k}^{\left(t\right)}$):

$$n_{l.k}^{\left(t\right)}∼N\text{Binom}\left(\mu_{\mathrm{lk}}^{\left(t\right)},N\right)$$

$$\begin{array}{c} E[n_{l.k}^{\left(t\right)}]\\ \;=\mu_{\mathrm{lk}}^{\left(t\right)}\\ \;=\exp(\beta_{0p[l]k}+\beta_{1k}B3{\text{yr}}_{\mathrm{lt}}+\beta_{2k}B1{\text{yr}}_{\mathrm{lt}}+\beta_{3k}{\text{DE}}_{l}+\beta_{4k}Y_{t}+\beta_{5k}\left(Y_{t}\times B3{\text{yr}}_{\mathrm{lt}}\right)\\ \;\;+\beta_{6k}\left(Y_{t}\times B1{\text{yr}}_{\mathrm{lt}}\right)+\beta_{7k}\left({\text{DE}}_{l}\times B3{\text{yr}}_{\mathrm{lt}}\right)+\beta_{8k}\left({\text{DE}}_{l}\times B1{\text{yr}}_{\mathrm{lt}}\right)) \end{array}$$

In this expression, $\beta_{0p[l]k}$ is a plot‐specific intercept and $\beta_{1k},\ldots ,\beta_{8k}$ are the regression slope parameters for group *k*. As for the covariates, $B3{\text{yr}}_{\mathrm{lt}}$ and $B1{\text{yr}}_{\mathrm{lt}}$ are binary variables denoting if transect *l* in year *t* received the low or high fire frequency treatments, respectively; ${\text{DE}}_{l}$ is the distance of transect *l* to the edge of the forest and $Y_{t}$ is the year at time *t*. Finally, $Y_{t}\times B3{\text{yr}}_{\mathrm{lt}}$ and $Y_{t}\times B1{\text{yr}}_{\mathrm{lt}}$ are interaction terms between year and treatments, allowing for the impact of fires to change with time. Similarly, ${\text{DE}}_{l}\times B3{\text{yr}}_{\mathrm{lt}}$ and ${\text{DE}}_{l}\times B1{\text{yr}}_{\mathrm{lt}}$ are interaction terms between distance to edge and treatments, allowing for the effect of fire to be different depending on the distance to the forest edge.

For the “Block” experiment, we adopted the following regression structure:

$$n_{l.k}^{\left(t\right)}∼N\text{Binom}\left(\mu_{\mathrm{lk}}^{\left(t\right)},N\right)$$

$$E\left[n_{l.k}^{\left(t\right)}\right]=\mu_{\mathrm{lk}}^{\left(t\right)}=\exp\left(\beta_{0l}+\beta_{1}\text{Fire}1_{\mathrm{lt}}+\beta_{2}\text{Fire}2_{\mathrm{lt}}+\beta_{3}{\text{FA}}_{\mathrm{lt}}\right)$$

where $\beta_{0l}$ is a plot‐specific intercept. In this expression, $\text{Fire}1_{\mathrm{lt}},\text{Fire}2_{\mathrm{lt}}$, and ${\text{FA}}_{\mathrm{lt}}$ are binary variables denoting if plot *l* in year *t* was burned once, was burned twice, and if fuel was added, respectively.

For all models, slope parameters are deemed to be statistically significant and highly statistically significant if $\min(p\left(\beta_{p}<0\right),p\left(\beta_{p}>0\right))$ is smaller than 0.05 and 0.01, respectively. Finally, we define the characteristic species in each group as those that are more than twice as abundant in the focus group when compared to the other groups.

## 3 RESULTS

### 3.1 Simulation results

**FIGURE 2:**
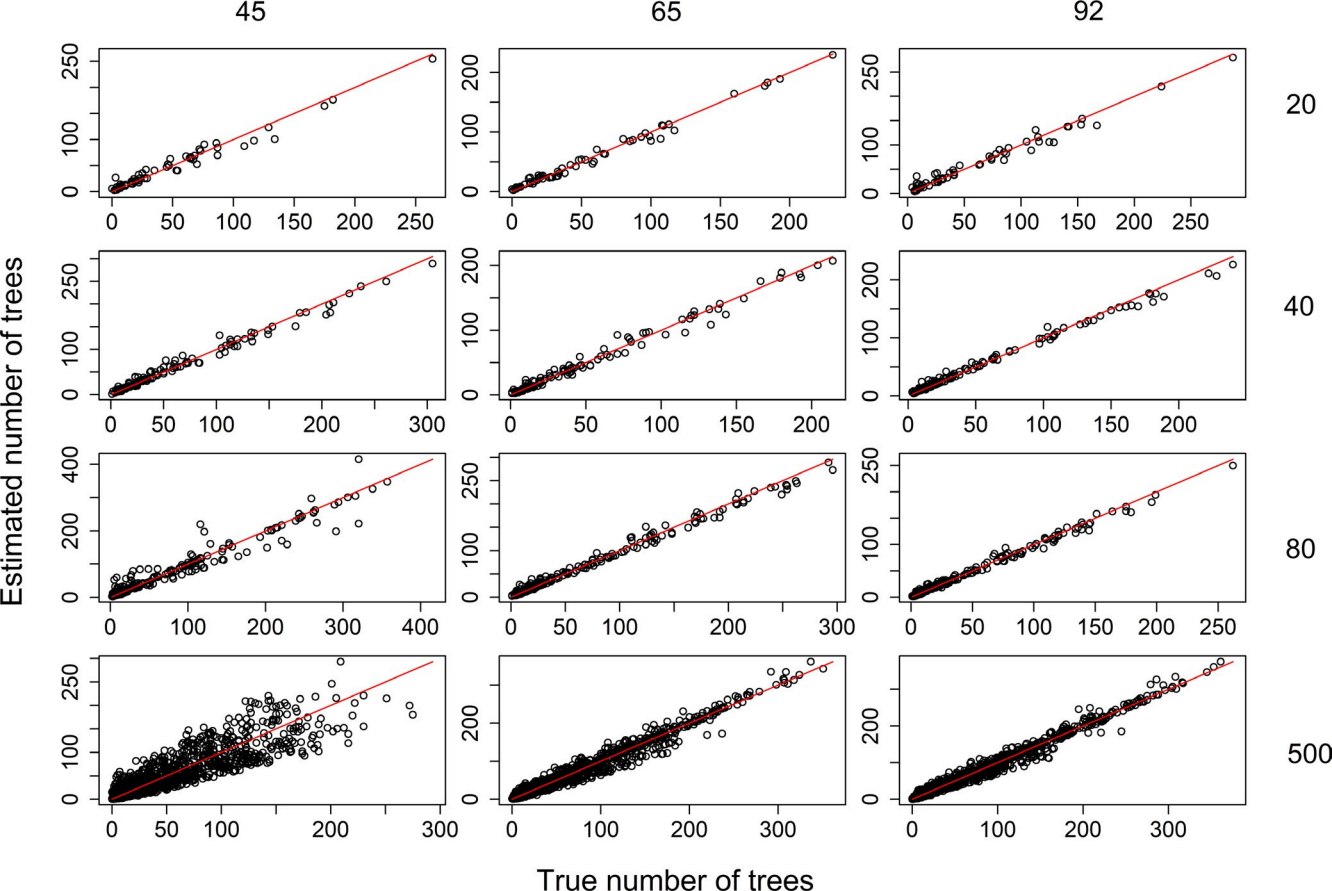
LDAcov is able to estimate well the number of individuals in each group and location ($n_{l.k}$) for different scenarios regarding number of species and locations. True and estimated values for $n_{l.k}$ are displayed in the *x*‐ and *y*‐axes, respectively. The 1:1 line is shown in red. Left to right panels display results of scenarios where the number of species is equal to 45, 65, and 92, respectively. Top to bottom panels display results of scenarios where the number of sites is equal to 20, 40, 80, and 500 locations, respectively

We find that our first‐stage model (i.e., the unconstrained LDA model) was able to correctly identify the existence of three groups (out of a maximum of 10 groups) of individuals in all 12 scenarios (Appendix S3). Furthermore, the second‐stage model (i.e., LDAcov) was able to estimate well all the parameters across all scenarios, including the number of individuals in each group and location $n_{l.k}$ (Figure 2) and the coefficients $\beta_{k}$ (Appendix S3). Importantly, our two‐stage approach consistently performed better than the approach that fits all parameters at once (Appendix S3).

### 3.2 Big‐plot experiment

**FIGURE 3:**
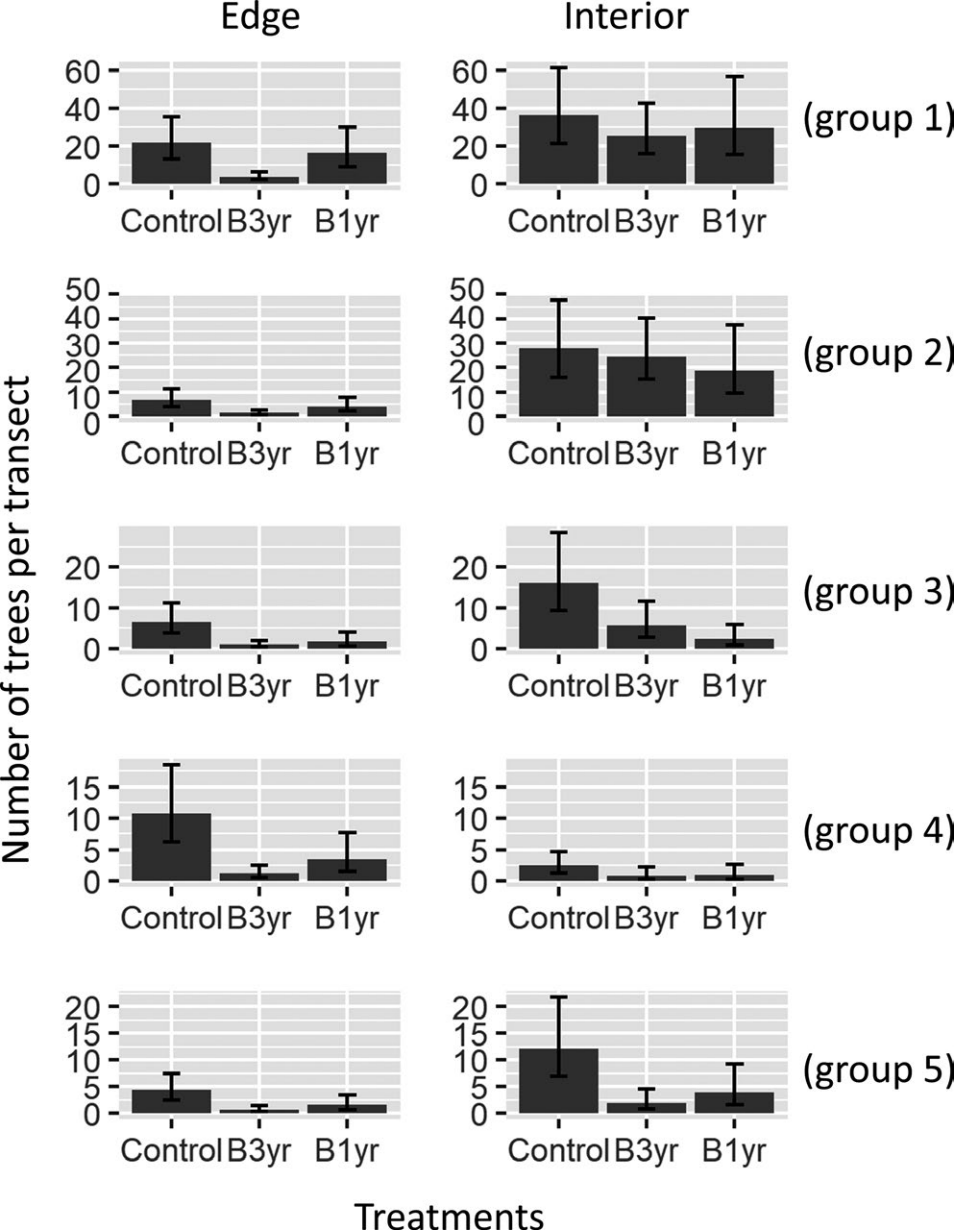
Model predictions of the number of trees per transect for each group and each treatment by the end of the “Big‐plot” experiment (i.e., 2016). These predictions were made for the plot with B3yr. Left and right panels correspond to predictions for the forest edge and forest interior transects, respectively. Treatments refer to no fire (‘Control’), fire approx. every 3 years (‘B3yr’), and fire approx. every year (‘B1yr’). Error bars are 95% credible intervals

The first‐stage model identified five main groups, representing approximately 97% of all the trees. Based on LDAcov with 5 groups, we found several of the covariates to be statistically significant (Table 1). For example, we found that as distance to edge increased, the abundance of groups 1, 2, 3, and 5 tended to increase whereas the abundance of group 4 decreased. These patterns suggest that group 4 is more characteristic of forest edges whereas the other groups are much more common in the forest interior. This is clearly depicted by comparing the control results for the forest edge to those from the forest interior (Figure 3).

**TABLE 1:** Estimated slope parameters for each group

Variable	Group				
	1	2	3	4	5
Distance to edge	0.19*	0.51**	0.33**	−0.54**	0.38**
B3yr (fire every 3 years)	−0.41*	−0.38*	−0.58**	−0.8**	−0.69**
B1yr (fire almost every year)	−0.02	−0.08	−0.64**	−0.64**	−0.41*
Year	0.52**	0.51**	0.29*	0.4*	0
Interaction: Edge × B3yr	0.03	0.05	−0.21	0.07	−0.06
Interaction: Edge × B1yr	−0.59**	−0.46**	−0.67**	−0.69**	−0.78**
Interaction: Year × B3yr	−0.16	−0.28*	−0.63**	−0.3	−0.44*
Interaction: Year × B1yr	−0.23**	−0.31**	−0.28**	−0.11	−0.19*

Note

The symbols * and ** represent significant and highly significant results, respectively.

The fire treatments tended to decrease the abundance of all groups (Table 1). The exception to this pattern was the weak effect of the annual fires (B1yr) on groups 1 and 2 (Table 1). Parameter estimates for B3yr were larger in magnitude than those for B1yr, except for group 3, indicating that fire in the B3yr treatment had a more severe negative impact on the abundance of groups when compared to B1yr, probably a consequence of substantial fuel buildup within these 3‐year time intervals.

Whenever significant, the interaction between distance to forest edge and fire was positive, suggesting that the negative effects of fire were less pronounced the farther trees were from the edge of the forest. These results reveal the synergistic effect between fragmentation and fire effects on tree mortality. Finally, the abundance of all groups was generally declining with time even in the control group but, as revealed by the significant interaction with fire for many of these groups, this decline with time was substantially exacerbated by fire. Interestingly, the parameters associated with the interaction between year and B3yr were consistently significant and greater in magnitude when compared to the equivalent parameters for B1yr, reinforcing the hypothesis that infrequent fires can be more damaging than annual fires (Balch et al., 2008).

The characteristic species in each group conform to what we expected (see details in Appendix S4). For instance, among the characteristic species of each group, the highest proportion of pioneer species was found in the group that was more abundant at the edge of the forest (i.e., group 4). In particular, three of the characteristic species of group 4 were *Mabea fistulifera*, *Cecropia* *palmate*, and *Schefflera morototoni*, all of which are commonly found along forest edges, in early successional states or in open habitats (Lorenzi, 2000; Sposito & Santos, 2001). Similarly, the characteristic species of groups 1 and 2 tended to have thicker bark than the characteristic species from groups 3–5 (see Appendix S4), potentially explaining why these two groups were more resistant to annual fires.

### 3.3 “Block” experiment

**FIGURE 4:**
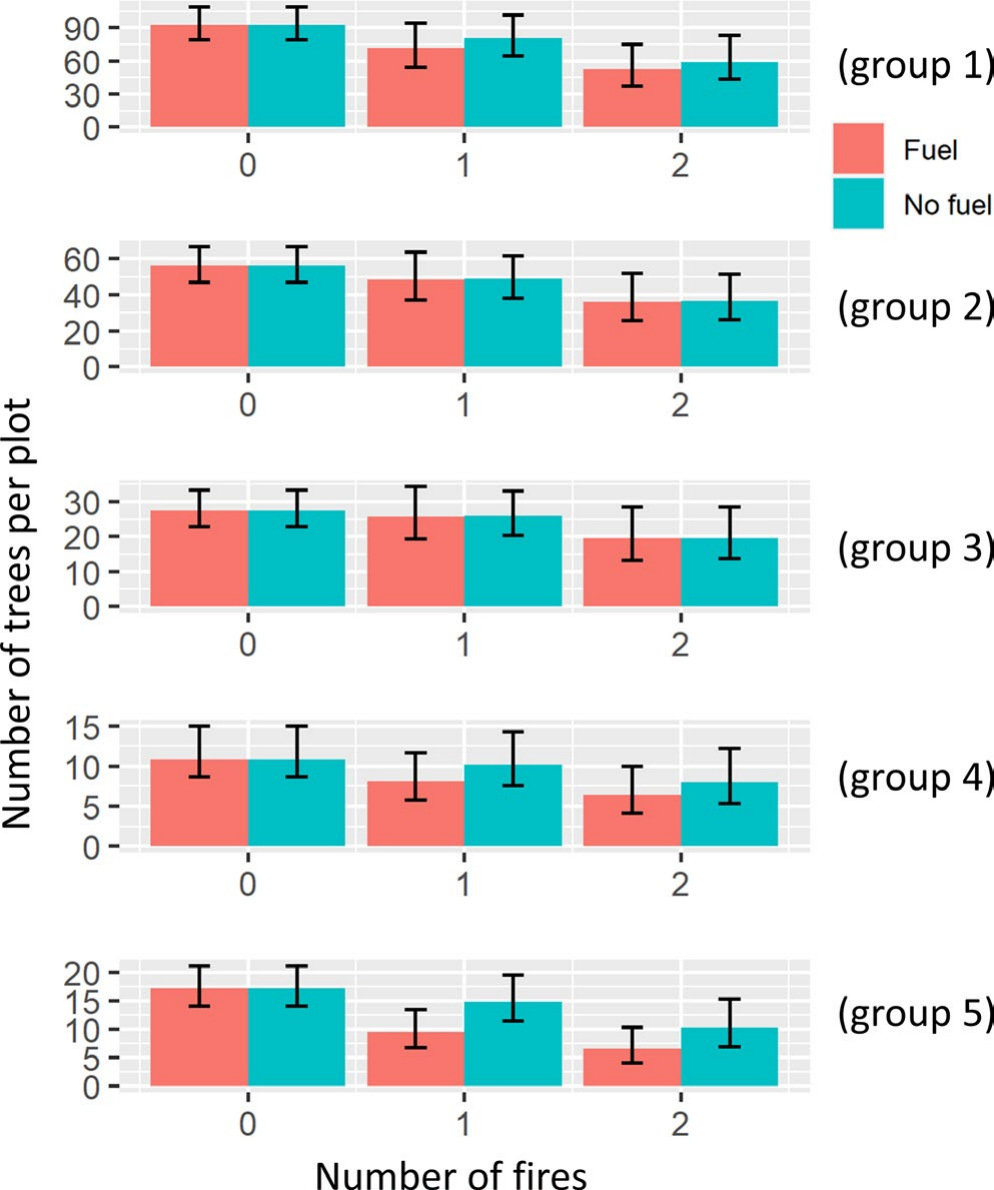
Predicted number of trees per plot for different numbers of fire (*x*‐axis), with (red) and without fuel addition (green). Predictions were made for the baseline plot (i.e., plot 1). Error bars are 95% credible intervals. Notice that we assume that fuel addition does not alter the control treatment. For this reason, results for zero fires with and without fuel addition are identical

The model without any covariates also identified five main groups, representing approximately 95% of all the trees. Based on 5 groups, the LDAcov model revealed that, while the first fire seems to have decreased the abundance across all groups, these effects were not significant. On the other hand, the parameters associated with the second fire were generally more negative than those from the first fire, with significant effects observed for groups 1, 2, and 5 (Table 2). These results suggest that the second fire was substantially more severe than the first fire, perhaps as a result of trees already being weakened by the first fire. Finally, fuel addition generally tended to have a negative effect on abundance, but this effect was only statistically significant for group 5 (Figure 4).

**TABLE 2:** Estimated slope parameters for each group

Parameter	Group				
	1	2	3	4	5
Intercept	4.53**	4.03**	3.31**	2.39**	2.85**
First fire	−0.13	−0.14	−0.06	−0.07	−0.15
Second fire	−0.31*	−0.29*	−0.28	−0.24	−0.37*
Fuel addition	−0.12	−0.01	0	−0.23	−0.45**

Note

Significant and highly significant results are emphasized * and **, respectively.

The characteristic species in group 5 were all understory species (i.e., species that tended to have individuals with smaller diameter), which might explain why this was the only group that was affected by both the second fire and fuel addition (see details in Appendix S4). Furthermore, similar to the results found for the “Big‐plot” experiment, the characteristic species of groups 3 and 4 tended to have thicker bark when compared to the characteristic species of the other groups, helping to explain why these two groups were not significantly impacted by any of the fires or fuel addition (see Appendix S4).

## 4 DISCUSSION

In this article, we have described LDAcov, a novel model that can provide inference and prediction by embedding a regression structure within the standard LDA model. We illustrate the use of this model on data from two fire experiments in the Brazilian Amazon, enabling inference on how fragmentation and fire jointly affect species composition of these forests. It is important to note that, because LDAcov is a type of unsupervised method, it is much more challenging to evaluate the quality of its results when compared to supervised methods (James et al., 2013). For this reason, we validate LDAcov by determining how well its results based on the fire experiments data are corroborated by earlier findings using different methods.

### 4.1 Fire effects on plant assemblages

Based on the data from the “Big‐plot” experiment, our finding of increased impact on species composition associated with B3yr when compared to B1yr is corroborated by two important processes studied at the same experiment. The longer intervals between fires in the B3yr treatment enabled fuel buildup (Balch et al., 2015), and two of the fire events on the B3yr coincided with drought years (2007 and 2010) (Brando et al., 2014). More fuel in drier conditions resulted in increased burned area and higher fire intensity, particularly along the forest edge neighboring an agricultural field, ultimately leading to higher postfire tree mortality, higher losses in aboveground live biomass (Brando et al., 2014) and increased grass invasion (Silverio et al., 2013). Based on data from the “Block” experiment, we find that fuel addition tends to decrease the abundance of all groups, but that this effect was only significant for a single group with understory characteristic species. These results are corroborated by the finding that fuel addition resulted in the increased burned area and flame height, but not fireline intensity (Brando et al., 2016). Nevertheless, this experiment clearly reveals that, while a single understory fire might not substantially change species abundance, subsequent fires can have strong impact on plant assemblages, especially for small‐sized species and species with thin bark. Taken together, these results reveal the compounding impacts of multiple fire events and fragmentation, a scenario commonly found across the southern edge of Amazon (Brando, Paolucci, et al., 2019). The burned area in the region is projected to double in the next three decades (Brando et al., 2020), and the differential impacts of fire along forest edges on forest species composition can contribute to the degradation of these forests.

### 4.2 Comparison to other methods

One model that also incorporates covariates within LDA is called Structural Topic Model (STM). In STMs, a multinomial regression is embedded within LDA to enable the use of covariates (Mimno & McCallum, 2008; Roberts et al., 2016). LDAcov differs from STM in that it is specifically focused on modeling the number of individuals in each group rather than the proportion/prevalence of individuals in each group. This is an important feature for two reasons. First, modeling the number of individuals in each group enables straightforward interpretation of regression coefficients, an important characteristic for statistical inference. On the other hand, the coefficients from the multinomial logistic regression adopted by STMs are challenging to interpret as the relationship between the prevalence of a given group and a particular covariate depends on the slope parameter of all the other groups (see Appendix S5). Second, the number of individuals in each group is often the primary focus of ecological interest and can reveal effects that might be missed by modeling prevalence instead of abundance. For example, if fire reduces the abundance of trees in all groups equally, then the multinomial logistic regression described above would not detect a significant effect of fire because the prevalence of each group would remain the same. Similarly, if fire increases the prevalence of group 1 relative to group 2, it will not be clear if this happened because fire decreased the abundance of group 2 with no effect on group 1 or because fire increased the abundance of group 1 with no effect on group 2.

Other methods also exist that cluster plots and allow for covariates (Hill et al., 2020; Woolley et al., 2019). For example, a model that is somewhat similar to LDAcov is called the Regions of Common Profile (RCP) (Foster et al., 2017; Lyons et al., 2017). This is a type of mixture‐of‐regression model which groups sites that have similar species composition (hence the name regions of common profile). Within this model, a multinomial logistic regression enables covariates to influence the probability of each site being associated with a particular group. A key difference between LDAcov and RCP is that a site can only belong to a single group in RCP whereas LDAcov enables a plot to be comprised of multiple groups. This is important because, as illustrated in Valle et al. (2018), it implies that RCP will require more groups to fit the data equally well as LDA with fewer groups. Indeed, we have observed exactly this when we fitted RCP models (using the R package “RCPmod”) to our simulated data, regardless if the optimal number of groups was selected using AIC or BIC (see Appendix S3). Another important difference between LDAcov and RCP refers to the interpretability of the regression coefficients. The RCP model, similar to STM, relies on a multinomial logistic regression model and, as a result, its regression parameters are more challenging to interpret (see Appendix S5).

Another promising dimension reduction model is called Species Archetype Models (SAMs) (Dunstan et al., 2011, 2013). In these models, species are grouped according to how they respond to the covariates. We relied on the R package “ecomix” to fit SAMs. Within this package, first the optimal number of groups is identified using BIC and then uncertainty on regression parameters is estimated using a bootstrap approach based on the optimal model. Our experience has been that it can sometimes be challenging to fit these models. For example, to fit the “big plot” data, we varied the number of groups from 2 to 15 and we used the function “species_mix.multifit” to fit SAM 10 times for each number of groups. According to BIC, the optimal number of groups for these data was equal to 7. However, when examining more closely the results for the model fitted with 7 groups, we found that 3 groups were empty, suggesting that the algorithm did not find a good solution and resulting in numerical issues when estimating the uncertainty in the regression coefficients (e.g., standard errors and p‐values equal to zero).

Our perspective is that the development of novel multispecies models is an area of active research and that many of the existing models (e.g., SAMs and RCPs) can generate valuable insights despite having limitations. Importantly, we believe that LDAcov will be a useful addition to toolkit of ecologists interested in making community‐level inference. Future work on LDAcov could more explicitly incorporate spatial correlation, a feature that very few multispecies models include (see review in Norberg et al., 2019). Furthermore, the addition of species‐specific dispersion parameters in LDAcov (a feature that is implemented in a straightforward fashion in SAM) could be useful to allow for differences in spatial aggregation of different species. Finally, enabling LDAcov to accommodate for sampling artifacts (e.g., survey method, sampling effort, and season of data collection; similar to RCP) would probably be a very useful future extension for LDAcov.

Determining how anthropogenic stressors (e.g., timber logging, fire, and hunting) impact biodiversity is critical for an accurate picture of ecosystems services (e.g., carbon storage and water provisioning). However, assessing these impacts is particularly challenging for biodiversity‐rich system because of the large number of species, requiring methods that can reduce the dimensionality of the data while also making a statistically valid inference. The LDAcov was created to address this need. Together with an R package, we have added a tutorial providing step‐by‐step instructions regarding how to use LDAcov and interpret its results (Appendix S2). We believe that the proposed model will be useful for scientists interested in understanding and predicting how species composition of biodiversity‐rich ecosystems changes along environmental gradients, particularly for gradients that arise from large‐scale anthropogenic stressors (e.g., climate change, fire, forest fragmentation, and saltwater intrusion).

## CONFLICT OF INTEREST

The authors declare that they have no conflict of interest.

## AUTHOR CONTRIBUTIONS

**Denis Valle:** Conceptualization (lead); Formal analysis (lead); Funding acquisition (equal); Investigation (lead); Methodology (lead); Resources (equal); Software (lead); Visualization (lead); Writing‐original draft (lead); Writing‐review & editing (lead). **Gilson Shimizu:** Methodology (supporting); Software (equal). **Rafael** **Izbicki:** Methodology (supporting); Software (supporting). **Leandro** **Maracahipes:** Data curation (equal); Investigation (supporting); Writing‐review & editing (supporting). **Divino Vicente Silvério:** Data curation (equal); Investigation (supporting); Writing‐review & editing (supporting). **Lucas N**. **Paolucci:** Data curation (equal); Investigation (supporting); Writing‐review & editing (supporting). **Yusuf** **Jameel:** Methodology (supporting); Writing‐review & editing (supporting). **Paulo Brando:** Data curation (lead); Funding acquisition (equal); Investigation (supporting); Writing‐review & editing (supporting).

## Supporting information

Appendix S1

Appendix S2

Appendix S3

Appendix S4

Appendix S5

## Data Availability

The aggregate data for the fire experiments are stored and publicly available at Dryad (https://doi.org/10.5061/dryad.vq83bk3s5). The R package to run LDAcov is freely available at https://github.com/gilsonshimizu/ldacov.
