# Supplementary material for: The Latent Dirichlet Allocation model with covariates (LDAcov): A case study on the effect of fire on species composition in Amazonian forests
Source: Ecol Evol. 2021 May 5;11(12):7970–9. doi: 10.1002/ece3.7626 (PMC8216892; doi:10.1002/ece3.7626)
Supplement: Supplementary file 1 — Appendix S1 [file ECE3-11-7970-s005.docx]

**Appendix 1. Full conditional distribution for** $\boldsymbol{z}_{\boldsymbol{il}}$

In this derivation, we will be focused on location $l^{*}$ and species $s^{*}$. Suppose that, after we remove the i-th individual, we have $\left[ n_{l^{*}.1}^{\left( -i \right)},\ldots,n_{l^{*}.K}^{\left( -i \right)} \right]$. Furthermore, after removing the i-th individual, we also have $\left[ n_{l^{*}1k}^{\left( -i \right)},\ldots,n_{l^{*}s^{*}k}^{\left( -i \right)},\ldots,n_{l^{*}Sk}^{\left( -i \right)} \right]$ for each group k.

The probability that individual i in location $l^{*}$ will be assigned to a particular group (say group 1), given that this individual belongs to species $s^{*}$, is proportional to the following expressions:

$$p\left( z_{il^{*}}=1 | y_{il^{*}}=s^{*},\ldots\right)\propto Mult\left( \left[ n_{l^{*}11}^{\left( -i \right)},\ldots,n_{l^{*}s^{*}1}^{\left( -i \right)}+1,\ldots,n_{l^{*}S1}^{\left( -i \right)} \right] | n_{l^{*}.1}^{\left( -i \right)}+1,\boldsymbol{\phi}_{\boldsymbol{1}} \right)\times Mult\left( \left[ n_{l^{*}12}^{\left( -i \right)},\ldots,n_{l^{*}s^{*}2}^{\left( -i \right)},\ldots,n_{l^{*}S2}^{\left( -i \right)} \right] | n_{l^{*}2}^{\left( -i \right)},\boldsymbol{\phi}_{\boldsymbol{2}} \right)\times NB\left( n_{l^{*}.1}^{\left( -i \right)}+1 | \mu_{l^{*}1},N \right)NB\left( n_{l^{*}.2}^{\left( -i \right)} | \mu_{l^{*}2},N \right)$$

The expression described above can be substantially simplified in the following way:

$$\propto Mult\left( \left[ n_{l^{*}11}^{\left( -i \right)},\ldots,n_{l^{*}s^{*}1}^{\left( -i \right)}+1,\ldots,n_{l^{*}S1}^{\left( -i \right)} \right] | n_{l^{*}.1}^{\left( -i \right)}+1,\boldsymbol{\phi}_{\boldsymbol{1}} \right)NB\left( n_{l^{*}.1}^{\left( -i \right)}+1 | \mu_{l^{*}1},N \right)$$

$$\propto\left[ \frac{\left( n_{l^{*}.1}^{\left( -i \right)}+1 \right)!}{n_{l^{*}11}^{\left( -i \right)}!\ldots\left( n_{l^{*}s^{*}1}^{\left( -i \right)}+1 \right)!\ldots n_{l^{*}S1}^{\left( -i \right)}!}\phi_{11}^{n_{l^{*}11}^{\left( -i \right)}}\times\ldots\times\phi_{1s^{*}}^{n_{l^{*}s^{*}1}^{\left( -i \right)}+1}\times\ldots\times\phi_{1S}^{n_{l^{*}S1}^{\left( -i \right)}} \right]\times\left[ \frac{\Gamma\left( n_{l^{*}.1}^{\left( -i \right)}+1+N \right)}{\Gamma\left( N \right)\left( n_{l^{*}.1}^{\left( -i \right)}+1 \right)!}p_{l^{*}1}^{N}\left( 1-p_{l^{*}1} \right)^{\left( n_{l^{*}.1}^{\left( -i \right)}+1 \right)} \right]$$

$$\propto\left[ \frac{\left( n_{l^{*}.1}^{\left( -i \right)}+1 \right)}{\left( n_{l^{*}s^{*}1}^{\left( -i \right)}+1 \right)}\phi_{1s^{*}}Mult\left( \left[ n_{l^{*}11}^{\left( -i \right)},\ldots,n_{l^{*}s^{*}1}^{\left( -i \right)},\ldots,n_{l^{*}S1}^{\left( -i \right)} \right] | n_{l^{*}.1}^{\left( -i \right)},\boldsymbol{\phi}_{\boldsymbol{1}} \right) \right]\times\left[ \frac{\left( n_{l^{*}.1}^{\left( -i \right)}+N \right)}{\left( n_{l^{*}.1}^{\left( -i \right)}+1 \right)}\left( 1-p_{l^{*}1} \right)\times NB\left( n_{l^{*}.1}^{\left( -i \right)} | \mu_{l^{*}1},N \right) \right]$$

$$\propto\frac{\left( n_{l^{*}.1}^{\left( -i \right)}+1 \right)}{\left( n_{l^{*}s^{*}1}^{\left( -i \right)}+1 \right)}\phi_{1s^{*}}\frac{\left( n_{l^{*}.1}^{\left( -i \right)}+N \right)}{\left( n_{l^{*}.1}^{\left( -i \right)}+1 \right)}\left( 1-p_{l^{*}1} \right)=\frac{\left( n_{l^{*}.1}^{\left( -i \right)}+N \right)}{\left( n_{l^{*}s^{*}1}^{\left( -i \right)}+1 \right)}\phi_{1s^{*}}\left( 1-p_{l^{*}1} \right)$$

Because $z_{il^{*}}$ has to be in the set 1,…,K, this implies that we can sample it from a categorical distribution. The probabilities that define this categorical distribution can be obtained by normalizing the expression above:

$$p\left( z_{il}=k | y_{il}=s,\ldots\right)=\frac{\frac{\left( n_{l.k}^{\left( -i \right)}+N \right)}{\left( n_{\mathrm{ls}k}^{\left( -i \right)}+1 \right)}\phi_{\mathrm{ks}}\left( 1-p_{lk} \right)}{\sum_{c=1}^{K} \frac{\left( n_{l.c}^{\left( -i \right)}+N \right)}{\left( n_{\mathrm{lsc}}^{\left( -i \right)}+1 \right)}\phi_{\mathrm{cs}}\left( 1-p_{lc} \right)}$$
