# Supplementary material for: The Latent Dirichlet Allocation model with covariates (LDAcov): A case study on the effect of fire on species composition in Amazonian forests
Source: Ecol Evol. 2021 May 5;11(12):7970–9. doi: 10.1002/ece3.7626 (PMC8216892; doi:10.1002/ece3.7626)
Supplement: Supplementary file 2 — Appendix S2 [file ECE3-11-7970-s002.pdf]

Appendix 2. ldacov - A new Latent Dirichlet Allocation (LDA) formulation with covariates

ldacov (Shimizu, Izbicki, and Valle (2020)) is an R package that estimates the Latent Dirichlet Allocation (LDA) model with covariates using an MCMC algorithm. The model implemented in this package uses the number of elements in each cluster as the response variable. The logarithmic link function used to relate covariates to the response variable allows an easy interpretation of the regression coefficients.

Installation

Use the devtools package to install ldacov directly from github.

```
install_github("gilsonshimizu/ldacov")
```

The package can then be loaded using

```
library(ldacov)
```

Simulated data

To run the LDA model with covariates, we need a counting matrix y with dimension $l \times s$ (l is the number of locations, and s the number of species). We also need an matrix X of dimension $l \times (d+1)$ with a column of 1's for the intercept and the values for each of the d covariates. We simulate these data with the code given below:

```
rm(list=ls())
library('MCMCpack')
set.seed(21)

nloc=20 #number of locations
nspp=80 #number of species
ncommun=3 #number of groups
nparam=3 #number of parameters

#simulate phi matrix
gamma1=0.1
phi.true=phi=rdirichlet(ncommun,rep(gamma1,nspp))

#create design matrix
tmp=matrix(runif(nloc*nparam,min=-1,max=1),nloc,nparam)
colnames(tmp)=paste0('cov',1:3)
X=cbind(1,tmp) #add column of 1 for the intercept

#parameters
```

```
b0=log(runif(ncommun,min=20,max=40))
betas.true=betas=rbind(b0,diag(2,nparam))

#get means of N_lk
media.true=media=exp(X%*%betas);

#generate N_lk
nlk=matrix(NA,nloc,ncommun)
NBN=20
for (oo in 1:ncommun){
  nlk[,oo]=rnbinom(nloc,mu=media[,oo],size=NBN)
}
nlk.true=nlk

#generate actual observations y
nspp1=ncol(phi)
array.lsk=array(0,dim=c(nloc,nspp1,ncommun))
for (oo in 1:nloc){
  for (k in 1:ncommun){
    array.lsk[oo,,k]=rmultinom(1,size=nlk[oo,k],prob=phi[k,])
  }
}
y=apply(array.lsk,c(1,2),sum)
sim_data=list(y=y,X=X)
```

Optimal number of clusters

We start by finding the optimal number of clusters. Although this quantity could be chosen *a priori*, here we use an LDA model without covariates that relies on a truncated stick-breaking (TSB) prior to identify the number of clusters (Albuquerque, Valle, and Li (2019)). This model is implemented using the function `gibbs.LDA`. The arguments required for this function are:

- `y`: counting matrix containing the data (rows are locations and columns are species);
- `ncomm`: maximum number of clusters;
- `ngibbs`: number of iterations for the Gibbs sampler;
- `nburn`: number of iterations to be discarded as burn-in;
- `psi`: parameter for the prior Dirichlet distribution used for ϕ_k (the vector of probabilities that characterizes each cluster k); and
- `gamma`: parameter for the TSB prior used for θ_l (the vector of probabilities that characterizes each location l).

```
set.seed(1)
lda_no_covariates=gibbs.LDA(y=sim_data$y,
                            ncomm=10,
                            ngibbs=10000,
                            nburn=5000,
                            psi=0.01,
                            gamma=0.1)
```

This function outputs a list which contains the following elements:

- `llk`: posterior samples for the log-likelihood;

- `theta`: posterior samples for the Θ matrix containing the proportion of each cluster in each location;
- `phi`: posterior samples for the Φ matrix containing the proportion of each species within each cluster;
- `vmat`: posterior samples for the V matrix in the stick-breaking formulation, which is then used to derive the Θ matrix; and
- `array.lsk`: the last posterior sample for an array with the number of individuals in each location, species, and cluster.

We first assess convergence by inspecting the trace-plot of the log-likelihood. This plot, which only shows the results after the pre-specified burn-in period, suggests that the algorithm has converged:

```
plot(lda_no_covariates$llk,type='l',xlab='Iterations',ylab='Log-likelihood')
```

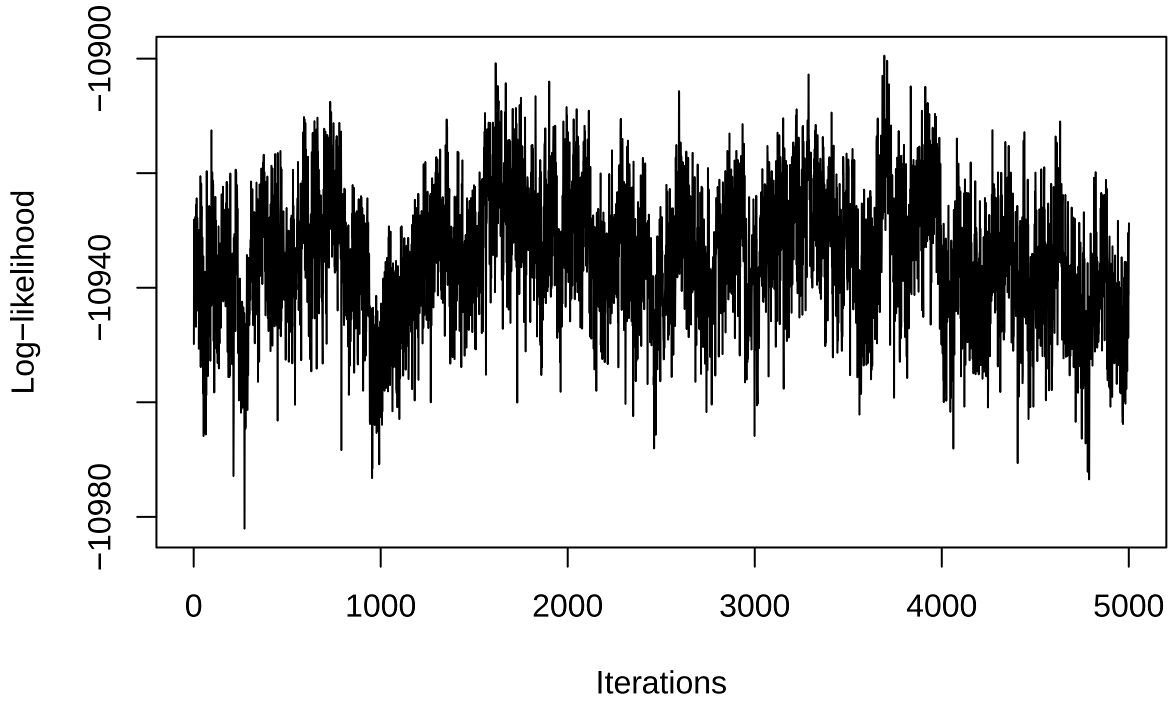

We used `array.lsk` to determine the optimal number of clusters (`theta` could have been used as well). On this dataset, the LDA model with the TSB prior seems to have identified 3 main cluster as these clusters contain more than 98% of the elements. Thus, we choose to use 3 clusters for the subsequent analysis.

```
array.lsk.init=lda_no_covariates$array.lsk
nlk=apply(array.lsk.init,c(1,3),sum)
theta=nlk/apply(nlk,1,sum)
colnames(theta)=paste0('Cluster',1:10)
rownames(theta)=paste0('Location',1:nrow(sim_data$y))
head(round(theta,2))
#>           Cluster1 Cluster2 Cluster3 Cluster4 Cluster5 Cluster6 Cluster7
#> Location1     0.24     0.65     0.08     0.03     0.00        0        0
#> Location2     0.25     0.45     0.27     0.03     0.00        0        0
#> Location3     0.28     0.18     0.50     0.03     0.01        0        0
```

```
#> Location4      0.57     0.40     0.03     0.00     0.00        0        0
#> Location5      0.42     0.09     0.46     0.03     0.00        0        0
#> Location6      0.09     0.57     0.33     0.00     0.00        0        0
#>            Cluster8 Cluster9 Cluster10
#> Location1        0        0         0
#> Location2        0        0         0
#> Location3        0        0         0
#> Location4        0        0         0
#> Location5        0        0         0
#> Location6        0        0         0

boxplot(theta,ylab=expression(theta),xlab='',ylim=c(0,1),las=2)
```

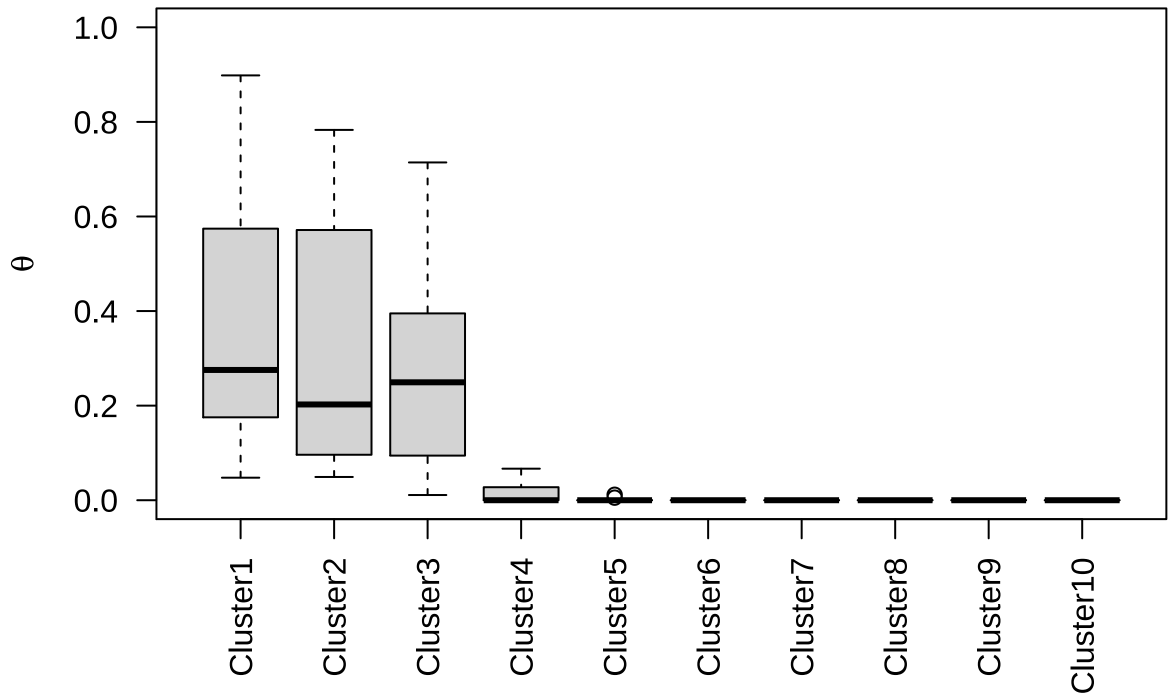


```
cumsum1=cumsum(colMeans(theta))
cumsum1[1:3]
#>  Cluster1  Cluster2  Cluster3
#> 0.3842006 0.7076556 0.9854716
```

Estimation using LDAcov

Once the number of clusters has been defined, the estimation of the parameters of ldacov are made through the function `gibbs.LDA.cov`. The arguments required by this function are:

- `ncomm`: number of clusters;

- `ngibbs`: number of iterations for the Gibbs sampler;
- `y`: counting matrix containing the data (rows are locations and columns are species);
- `xmat`: matrix containing covariate information (rows are locations and each column contains a different covariate). Notice that the first column of this matrix should be comprised of 1's if the regression model is to have an intercept;
- `phi.prior`: parameter to be used in the Dirichlet prior for Φ if this matrix is estimated;
- `array.lsk.init`: initial values for the `array.lsk` array;
- `var.betas`: variance parameters for the normal distribution priors for the regression coefficients;
- `phi.init`: posterior samples from the Φ matrix estimated by "gibbs.lda." If `estimate.phi` is TRUE, then these posterior samples are used simply to initialize the Φ matrix. If `estimate.phi` is FALSE, then these posterior samples are used instead of attempting to re-estimate the Φ matrix; and
- `estimate.phi`: if the Φ matrix is supposed to be estimated (TRUE or FALSE).

```
lda_with_covariates <- gibbs.LDA.cov(ncomm=3,
                                     ngibbs=1000,
                                     y=sim_data$y,
                                     xmat=sim_data$X,
                                     phi.prior=0.01,
                                     array.lsk.init=lda_no_covariates$array.lsk,
                                     var.betas=rep(100,ncol(sim_data$X)),
                                     phi.init=lda_no_covariates$phi,
                                     estimate.phi=FALSE)
```

This function outputs a list which contains the following elements:

- `llk`: posterior samples for the log-likelihood;
- `phi`: if `estimate.phi`=TRUE, then this consists of posterior samples for the Φ matrix containing the proportion of each species within each cluster. If `estimate.phi`=FALSE, then this just outputs random samples from lda_no_covariates$phi;
- `nlk`: posterior samples for the n_{lk} matrix containing the number of elements in each location l and cluster k;
- `betas`: posterior samples for the regression parameters for each cluster k;
- `fmodel`: posterior samples of the log-likelihood plus the log priors; and
- `NBN`: posterior samples for the dispersion parameter N from the negative-binomial regression.

One way to assess convergence consists of inspecting the trace-plot of the log-likelihood. This plot suggests that the algorithm has converged almost immediately. As a result, we discard just the first 100 iterations:

```
plot(lda_with_covariates$llk,type='l',xlab="iterations",ylab='log-likelihood')
```

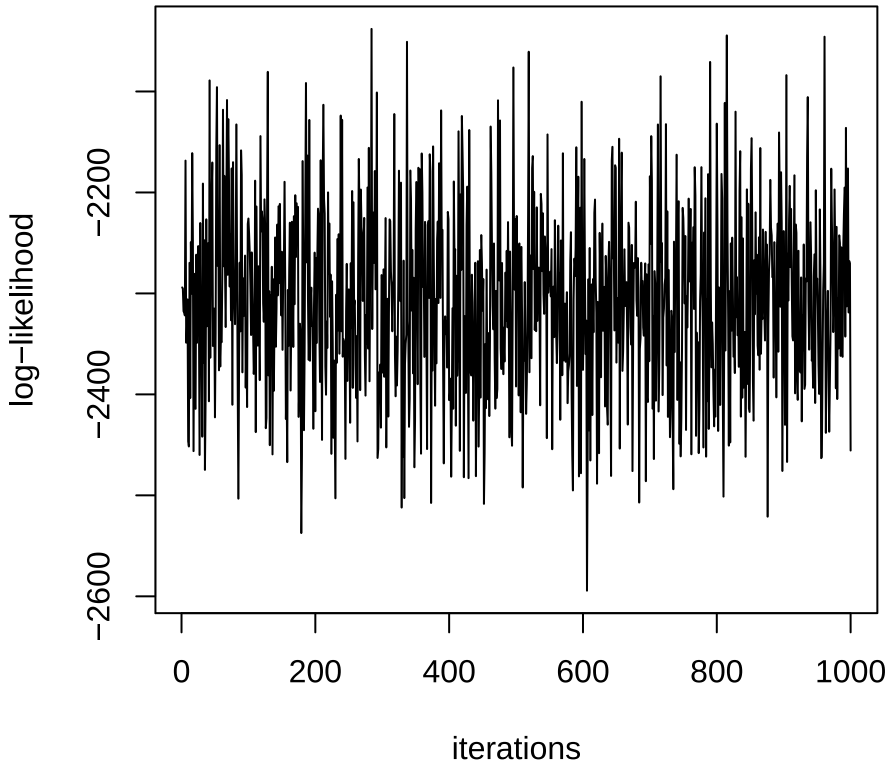

Typically, the main goals of the ldacov model are:

(i) to verify which covariates explain the quantities in each cluster,

(ii) to understand species distributions in each cluster (matrix Φ), and

(iii) to understand the distribution of the clusters within each location (matrix Θ).

We start by examining the proportion of each group in each location, given by Θ. We obtain the posterior mean for this matrix in the following way:

```
seq1=100:1000
tmp=matrix(colMeans(lda_with_covariates$nlk[seq1,]),nrow=nrow(sim_data$y),ncol=3)
theta <- tmp/rowSums(tmp)
colnames(theta)=paste0('Cluster',1:3)
rownames(theta)=paste0('Location',1:nrow(sim_data$y))
head(round(theta,2))
#>           Cluster1 Cluster2 Cluster3
#> Location1     0.27     0.64     0.09
#> Location2     0.27     0.48     0.25
#> Location3     0.34     0.20     0.46
#> Location4     0.53     0.43     0.04
#> Location5     0.48     0.11     0.41
#> Location6     0.12     0.59     0.29
```

Alternatively, we can examine directly the number of individuals estimated to be in each group at each location (n_{lk}). A comparison of the estimated and the true n_{lk} reveals that our algorithm is able to estimate well these latent variables.

```
nlk.estim=matrix(colMeans(lda_with_covariates$nlk[seq1,]),nrow=nrow(sim_data$y),ncol=3)
range1=range(c(nlk.estim,nlk.true))
order.comm=c(3,1,2) #re-order groups to match the order in the simulated data
plot(nlk.true,nlk.estim[,order.comm],xlim=range1,ylim=range1,xlab='True nlk',ylab='Estimated nlk')
lines(range1,range1,col='red')
```

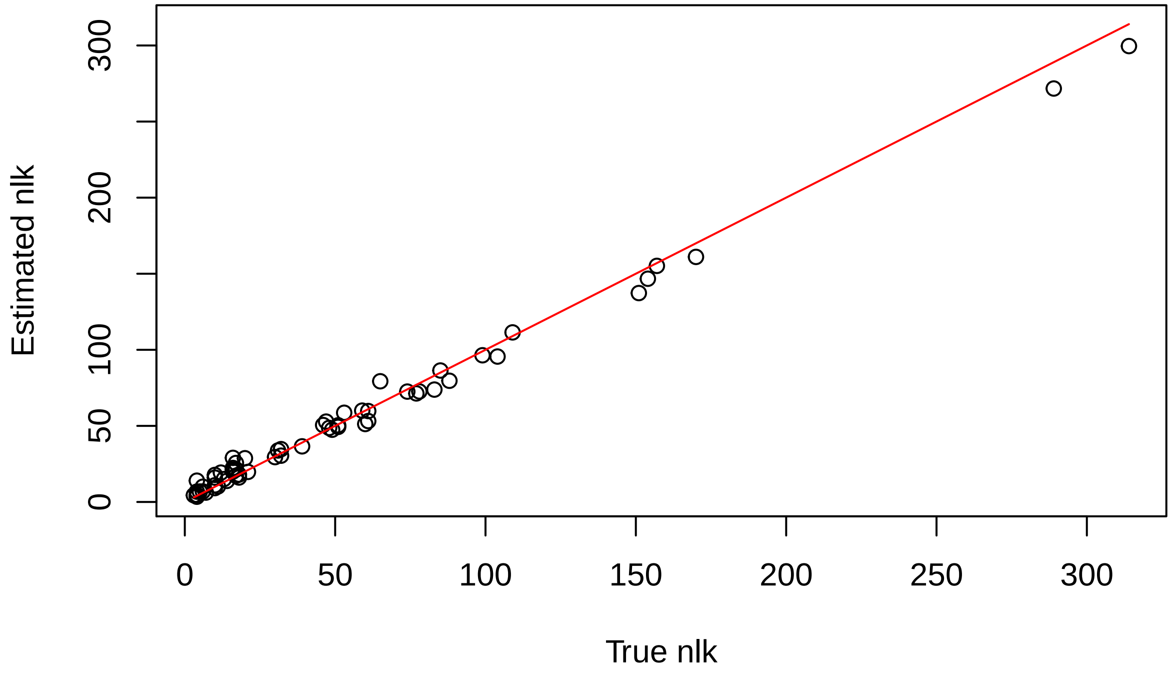

Similarly, we can also examine the Φ matrix. We calculate the posterior mean in the following way:

```
phi <- matrix(colMeans(lda_with_covariates$phi[seq1,]),nrow=3,ncol=ncol(sim_data$y))
rownames(phi)=paste0('Cluster',1:3)
colnames(phi)=paste0('Species',1:ncol(sim_data$y))
head(round(phi[,1:10],2))
#>          Species1 Species2 Species3 Species4 Species5 Species6 Species7
#> Cluster1     0.00     0.00     0.00     0.05     0.00     0.00     0.06
#> Cluster2     0.00     0.01     0.01     0.00     0.02     0.04     0.00
#> Cluster3     0.04     0.00     0.00     0.00     0.00     0.00     0.00
#>          Species8 Species9 Species10
#> Cluster1     0.00      0.1      0.00
#> Cluster2     0.00      0.0      0.00
#> Cluster3     0.02      0.0      0.01
```

A comparison of the estimated and the true Φ reveals that our method works well:

```
phi.estim <- phi[order.comm,]
range1=range(c(phi.estim,phi.true))
plot(phi.true,phi.estim,xlim=range1,ylim=range1,xlab='True Phi',ylab='Estimated Phi')
lines(range1,range1,col='red')
```

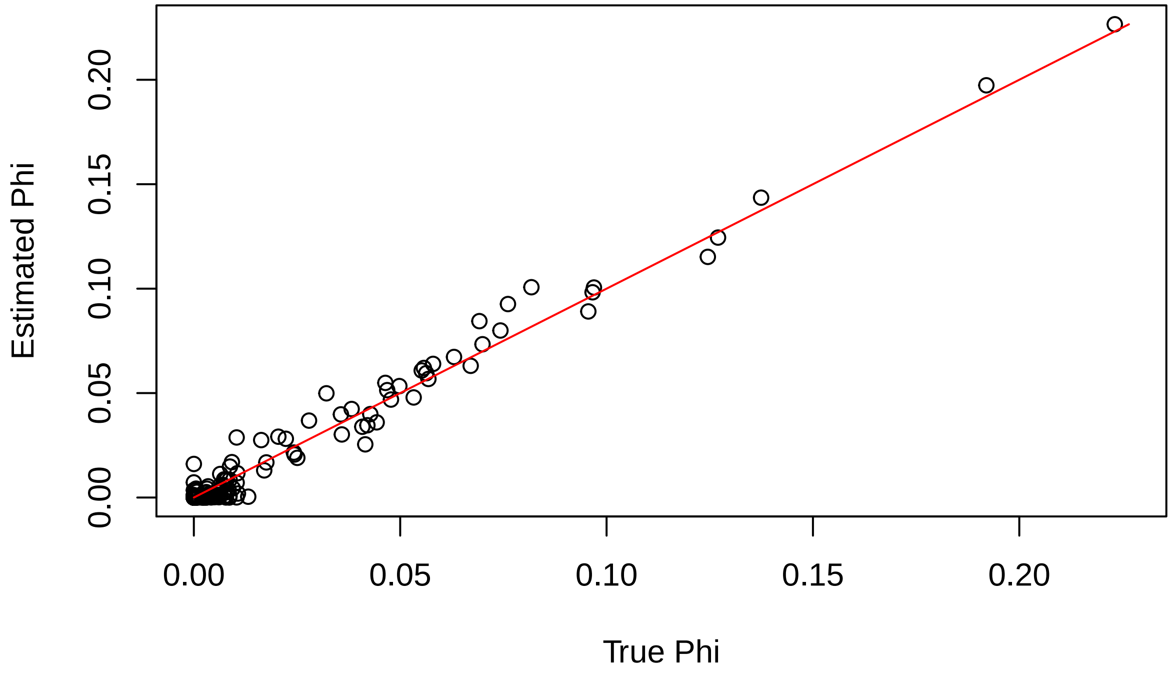

In many situations, it may be useful to identify the main cluster species. We define a species to be relevant for a given cluster if it appears at least twice more frequently in the focus cluster than in any other cluster. We use the following code to help us in this task:

```
phi.max=matrix(NA,nrow=3,ncol=ncol(sim_data$y))
for (i in 1:3){phi.max[i,]=apply(phi[-i,], 2, max)}
results=phi/phi.max
colnames(results)=colnames(phi)
rownames(results)=rownames(phi)
head(round(results[,1:10],2))
#>          Species1 Species2 Species3 Species4 Species5 Species6 Species7
#> Cluster1     0.00     0.06     0.00   212.94     0.00      0.0  3528.99
#> Cluster2     0.01     9.79   156.40     0.00   211.64   1000.2     0.00
#> Cluster3   147.54     0.10     0.01     0.00     0.00      0.0     0.00
#>          Species8 Species9 Species10
#> Cluster1      0.0  1433.73      0.22
#> Cluster2      0.0     0.00      0.00
#> Cluster3    383.6     0.00      4.61
```

For example, these results suggest that “Species 1” is 148 times more common in “Cluster 3” than in any

other cluster. Similarly, "Species 2" is 10 times more common in "Cluster 2" than in any other cluster. We use the following code to identify the main cluster species:

```
max_categ_cluster=apply(phi/phi.max, 1, function(x) names(sort(x,decreasing=TRUE)))
head(max_categ_cluster)
#>      Cluster1    Cluster2    Cluster3
#> [1,] "Species7"  "Species50" "Species27"
#> [2,] "Species19" "Species63" "Species72"
#> [3,] "Species33" "Species78" "Species71"
#> [4,] "Species9"  "Species6"  "Species24"
#> [5,] "Species17" "Species18" "Species8"
#> [6,] "Species16" "Species48" "Species51"
```

Finally, the posterior mean of the regression coefficients are extracted in the following way:

```
tmp=colMeans(lda_with_covariates$betas[seq1,])
betas=matrix(tmp,ncol=3)
colnames(betas)=paste0('Cluster',1:3)
rownames(betas)=paste0('Coefficients',1:nrow(betas))
head(round(betas,3))
#>               Cluster1 Cluster2 Cluster3
#> Coefficients1    3.809    3.826    3.257
#> Coefficients2    0.088    0.062    1.766
#> Coefficients3    1.527    0.145    0.222
#> Coefficients4    0.066    1.692    0.195
```

A comparison of the estimated and the true β_k reveals that our method works well:

```
betas.estim <- betas[,order.comm]
range1=range(c(betas.estim,betas.true))
plot(betas.true,betas.estim,xlim=range1,ylim=range1,xlab='True Betas',ylab='Estimated Betas')
lines(range1,range1,col='red')
```

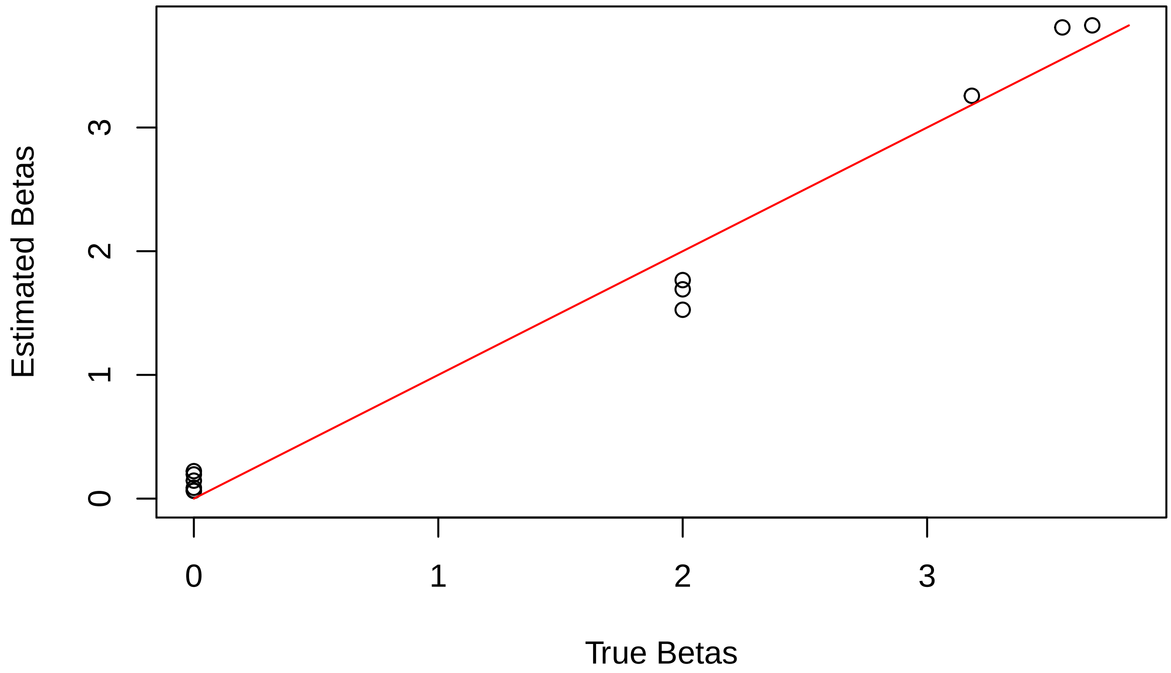

References

Albuquerque, Pedro HM, Denis Ribeiro do Valle, and Daijiang Li. 2019. "Bayesian LDA for Mixed-Membership Clustering Analysis: The Rlda Package." *Knowledge-Based Systems* 163: 988–95.

Shimizu, Gilson Yuuji, Rafael Izbicki, and Denis Ribeiro do Valle. 2020. "A New LDA Formulation with Covariates."
