# Supplementary material for: The Latent Dirichlet Allocation model with covariates (LDAcov): A case study on the effect of fire on species composition in Amazonian forests
Source: Ecol Evol. 2021 May 5;11(12):7970–9. doi: 10.1002/ece3.7626 (PMC8216892; doi:10.1002/ece3.7626)
Supplement: Supplementary file 3 — Appendix S3 [file ECE3-11-7970-s003.docx]

**Appendix 3. Additional simulation results**

Here we show the results for the number of groups based on our first-stage model. Recall that we assume a maximum of 10 groups (K=10) when running this model. However, for all simulated datasets, the three first groups accounted for > 97% of all individuals, strongly suggesting the existence of only three groups.


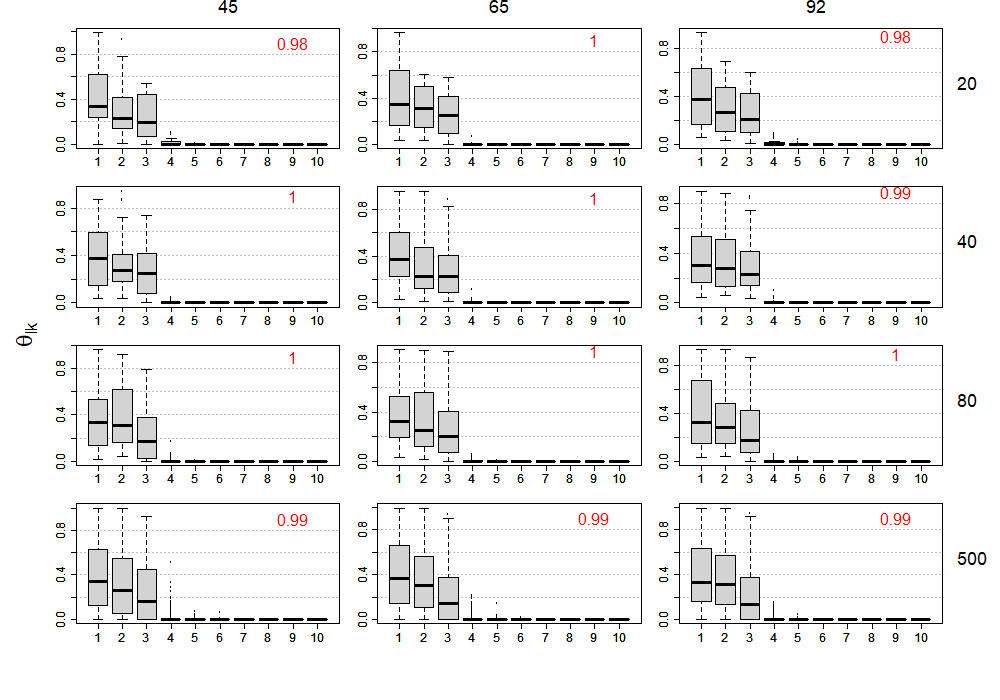


Fig. 1. Distribution of the estimated proportion $\theta_{lk}$ (y-axis) of each group (x-axis) based on the simulated data. Red numbers in upper right corner of each panel are the proportion of individuals estimated to be in the first three groups. Left to right panels display results of scenarios where the number of species is equal to 45, 65, and 92, respectively. Top to bottom panels display results of scenarios where the number of sites is equal to 20, 40, 80 and 500 locations, respectively.

Based on the results from the first-stage model, LDAcov was able to accurately estimate the parameters, as shown in the comparison of the estimates for the regression coefficients ($\boldsymbol{\beta}_{\boldsymbol{k}}$) and the species composition of each group ($\boldsymbol{\phi}_{\boldsymbol{k}}$) against the true parameter values for each scenario (Figures 2 and 3, respectively).


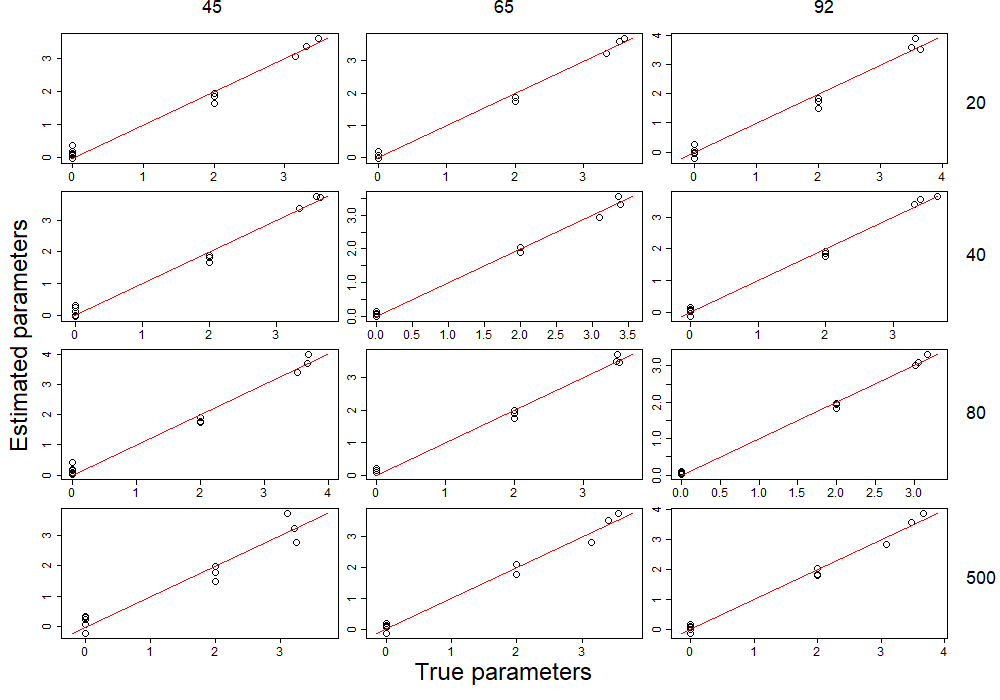


Fig. 2. LDAcov is able to estimate well the regression coefficients ($\boldsymbol{\beta}_{\boldsymbol{k}}$) for different scenarios regarding number of species and locations. True and estimated values for $\boldsymbol{\beta}_{\boldsymbol{k}}$ are displayed in the x and y axes, respectively. The 1:1 line is shown in red. Left to right panels display results of scenarios where the number of species is equal to 45, 65, and 92, respectively. Top to bottom panels display results of scenarios where the number of sites is equal to 20, 40, 80 and 500 locations, respectively.


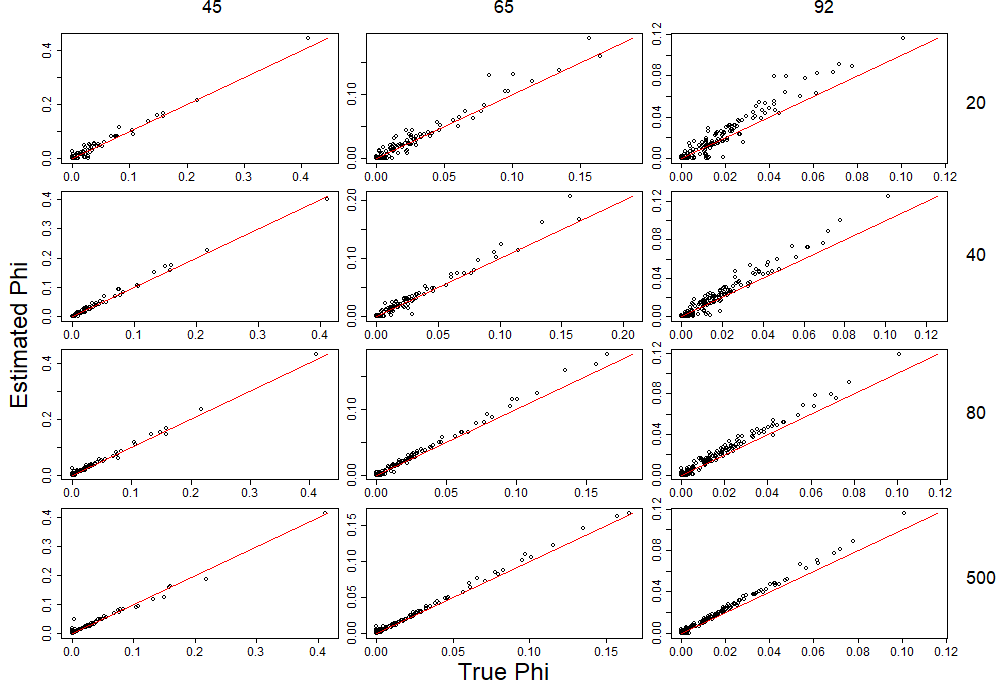


Fig. 3. LDAcov is able to estimate well the species composition of each group ($\boldsymbol{\phi}_{\boldsymbol{k}}$) for different scenarios regarding number of locations and number of species. True and estimated values for $\boldsymbol{\phi}_{\boldsymbol{k}}$ are displayed in the x and y axes, respectively. The 1:1 line is displayed in red. Left to right panels display results of scenarios where the number of species is equal to 45, 65, and 92, respectively. Top to bottom panels display results of scenarios where the number of sites is equal to 20, 40, 80 and 500 locations, respectively.

Using the simulated data, we also compare the proposed two-stage model (“2-stage”) with a model which simultaneously estimates all parameters (“Simultan.”). This comparison consistently reveals that the two-stage model can better estimate all parameters when compared to the “Simultan.” model (Table 1).

Table 1. The two-stage LDAcov model (“2-stage”) better estimates the parameters than the corresponding one-stage LDAcov model (“Simultan.”). Values correspond to mean squared error (MSE) comparing the estimated and true parameter values. Lower MSE indicate better results and are highlighted in bold. MSE for $\boldsymbol{\phi}_{\boldsymbol{k}}$ where re-scaled by multiplying by 1,000 for clarity.

| Number of locations | Number of species | $n_{l.k}$ | | $\boldsymbol{\beta}_{\boldsymbol{k}}$ | | $\boldsymbol{\phi}_{\boldsymbol{k}}$ | |
| --- | --- | --- | --- | --- | --- | --- | --- |
|  |  | 2-stage | Simultan. | 2-stage | Simultan. | 2-stage | Simultan. |
| 20 | 80 | **87** | 2153 | **0.03** | 0.67 | **0.06** | 1.81 |
| 20 | 160 | **34** | 1484 | **0.02** | 0.45 | **0.04** | 0.47 |
| 20 | 320 | **87** | 2489 | **0.05** | 0.64 | **0.04** | 0.13 |
| 40 | 80 | **70** | 3213 | **0.03** | 0.69 | **0.02** | 1.75 |
| 40 | 160 | **39** | 1195 | **0.01** | 0.45 | **0.04** | 0.46 |
| 40 | 320 | **33** | 1907 | **0.01** | 0.49 | **0.02** | 0.14 |
| 80 | 80 | **395** | 4130 | **0.04** | 0.71 | **0.02** | 1.81 |
| 80 | 160 | **50** | 3370 | **0.02** | 0.71 | **0.01** | 0.60 |
| 80 | 320 | **21** | 1127 | **0.01** | 0.41 | **0.01** | 0.10 |
| 500 | 80 | **470** | 1701 | **0.11** | 0.75 | **0.05** | 1.77 |
| 500 | 160 | **120** | 2586 | **0.03** | 0.72 | **0.01** | 0.61 |
| 500 | 320 | **80** | 2471 | **0.02** | 0.72 | **0.01** | 0.21 |

Finally, although LDAcov always identified the 3 groups used to simulate the data, we find that Regions of Common Profile (RCP) model typically identifies many more groups, regardless if the optimal number of groups is determined using AIC or BIC (Table 2).

Table 2. Optimal number of groups identified by RCP based on AIC or BIC, when fitted to the simulated data.

| Number of locations | Number of species | Number of groups estimated by RCP | |
| --- | --- | --- | --- |
|  |  | AIC | BIC |
| 20 | 45 | 4 | 3 |
| 20 | 65 | 4 | 3 |
| 20 | 92 | 4 | 2 |
| 40 | 45 | 8 | 4 |
| 40 | 65 | 5 | 3 |
| 40 | 92 | 7 | 4 |
| 80 | 45 | 10 | 6 |
| 80 | 65 | 8 | 6 |
| 80 | 92 | 9 | 5 |
| 500 | 45 | 10 | 10 |
| 500 | 65 | 10 | 10 |
| 500 | 92 | 10 | 10 |
