# Supplementary material for: The Latent Dirichlet Allocation model with covariates (LDAcov): A case study on the effect of fire on species composition in Amazonian forests
Source: Ecol Evol. 2021 May 5;11(12):7970–9. doi: 10.1002/ece3.7626 (PMC8216892; doi:10.1002/ece3.7626)
Supplement: Supplementary file 4 — Appendix S4 [file ECE3-11-7970-s004.docx]

**Appendix 4. Additional results from the fire experiment datasets**

Table 1. Characteristics species of the groups identified by LDAcov based on the “big plot” experiment data.

| Groups | | | | |
| --- | --- | --- | --- | --- |
| 1 | 2 | 3 | 4 | 5 |
| Aspidosperma desmanthum | Aspidosperma obscurinervium | Bellucia grossularioides | Cecropia palmata | Aspidosperma excelsum |
| Emmotum fagifolium | Hirtella bicornis | Connarus perrotteti | Cordia bicolor | Enterolobium schomburgkii |
| Guatteria schomburgkiana | Miconia punctata | Himatanthus aticulatus | Erythroxylon rufum | Licania egleri |
| Humiria balsamifera | Miconia pyrifolia | Jacaranda copaia | Ficus unknow | Xylopia cayennensis |
| Hymenaea courbaril | Ouratea discophora | Miconia unknow | Licania gracilipes |  |
| Talisia cerasina | Pouteria ramiflora | Nectandra cuspidata | Mabea fistulifera |  |
|  | Sloanea eichleri | Ocotea cujumari | Mollia lepidota |  |
|  |  | Ormosia paraensis | Schefflera morototonii |  |
|  |  | Protium guianense | Tachigali vulgaris |  |
|  |  | Trattinnickia burserafolia | Simaruba amara |  |
|  |  | Xylopia frutescens | Tapirira guianensis |  |
|  |  | Zanthoxylum riedelianum |  |  |

Table 2. Characteristics species of the groups identified by LDAcov based on the “block” experiment data.

| Groups | | | | |
| --- | --- | --- | --- | --- |
| 1 | 2 | 3 | 4 | 5 |
| Aspidosperma obscurinervium | Bocageopsis mattogrossensis | Callichlamys latifolia | Byrsonima aerugo | Dacryodes microcarpa |
| Diplotropis purpurea | Doliocarpus spatulifolius | Cordia bicolor | Cheiloclinium cognatum | Hirtella glandulosa |
| Inga heterophylla | Maprounea guianensis | Elachyptera floribunda | Connarus perrotteti | Miconia punctata |
| Licania egleri | Matayba arborescens | Guatteria schomburgkiana | Erythroxylon rufum | Trattinnickia glaziovii |
| Mabea fistulifera | Myrcia multiflora | Himatanthus sucuuba | Himatanthus ryrifolia | Minquartia guianensis |
| Miconia gratissima | Ouratea discophora | Micropholis egensis | Hippocratea volubilis |  |
| Nectandra cuspidata | Vochysia vismiifolia | Miconia minutiflora | Mollia lepidota |  |
| Ormosia paraensis | Xylopia frutescens | Pseudolmedia murure | Ocotea cujumari |  |
| Pourouma velutina |  | Talisia cerasina | Parkia pendula |  |
| Pseudolmedia laevigata |  |  | Schefflera morototonii |  |
| Salacia impressifolia |  |  | Simaruba amara |  |
| Trichilia quadrijuga |  |  | Sloanea eichleri |  |
|  |  |  | Virola sebifera |  |
|  |  |  | Xylopia spp |  |


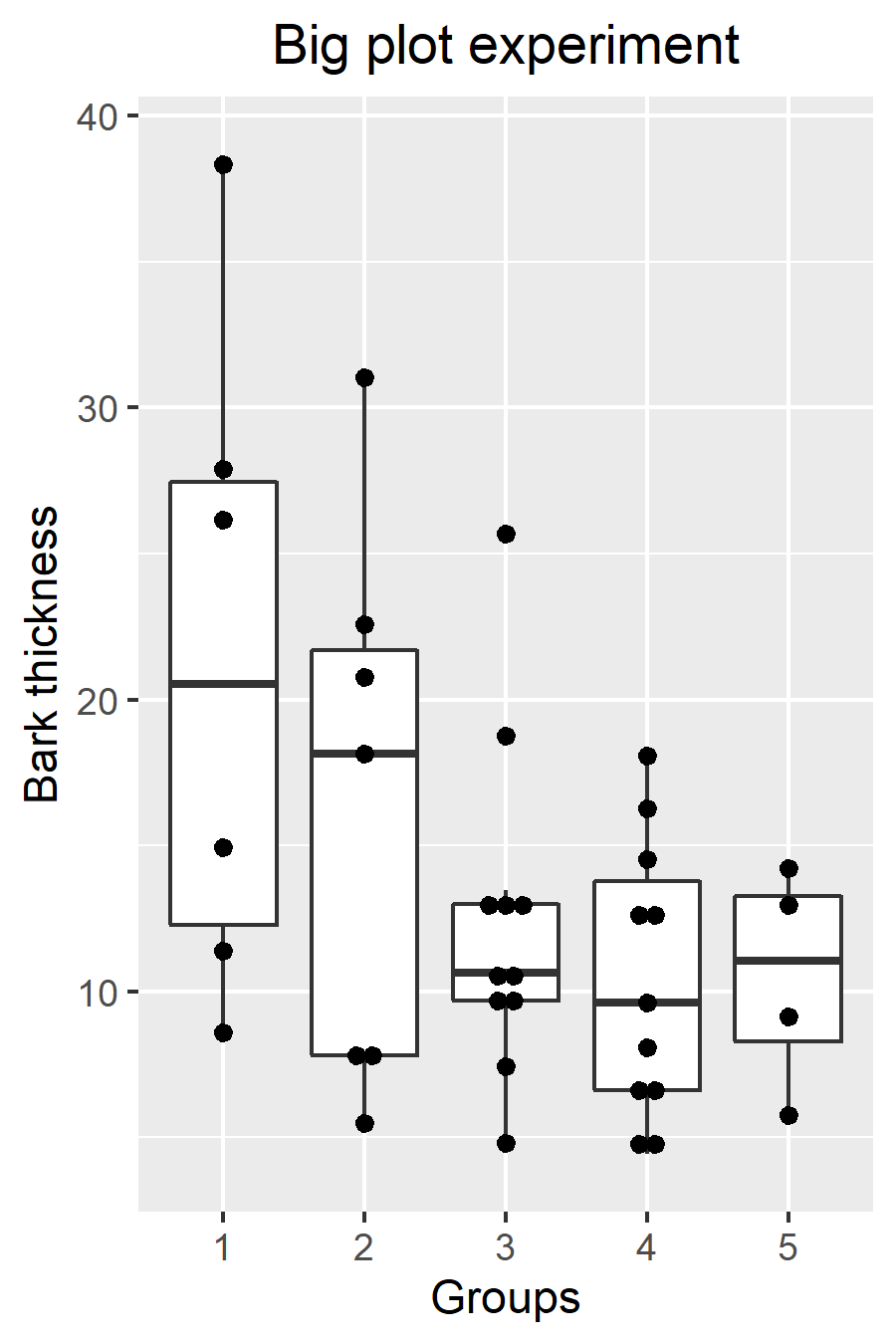

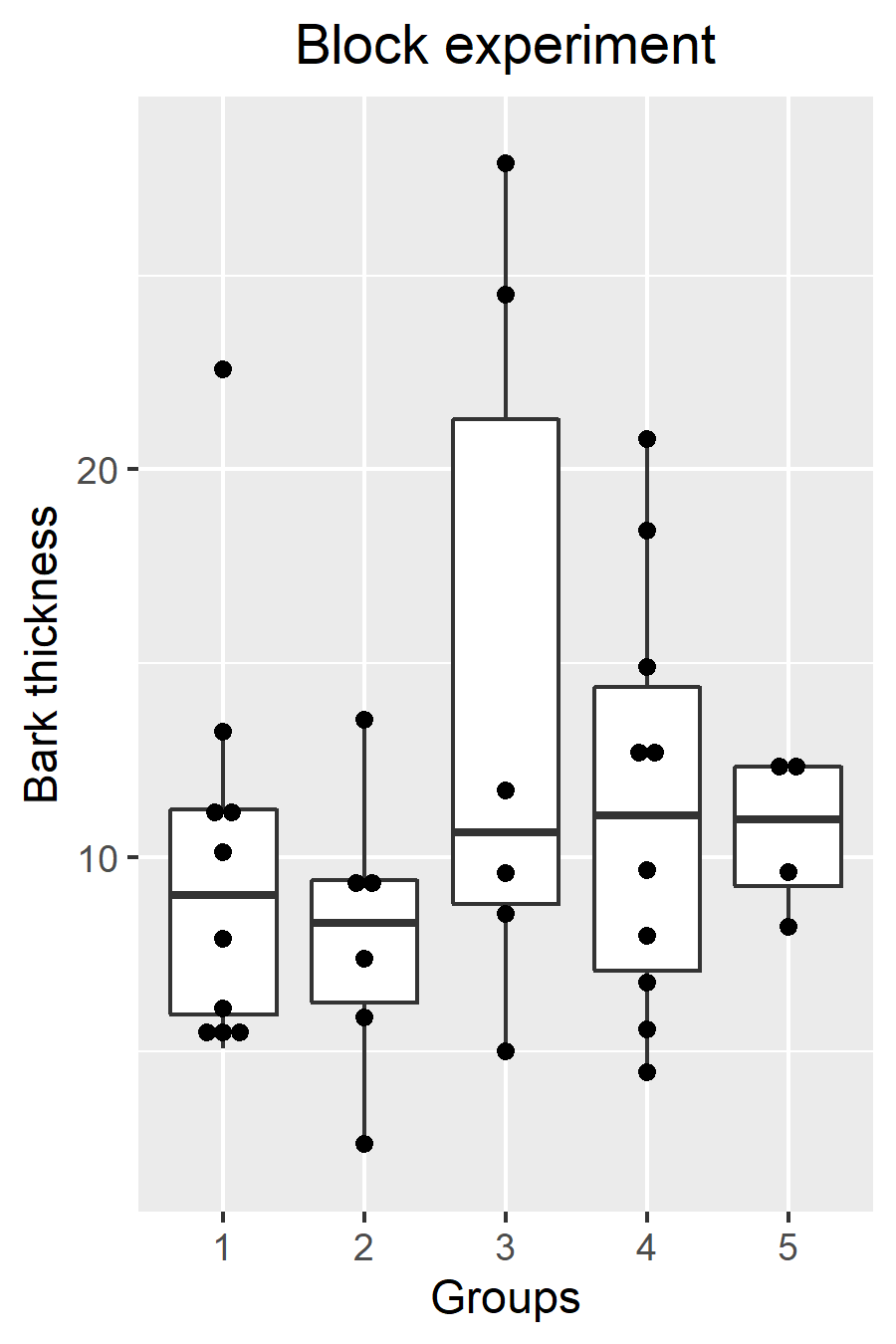


Fig. 1. Characteristic species from groups 1 and 2 from the “Big-plot” experiment (left panel) tended to have thicker bark when compared to the other groups. Similarly, characteristic species from groups 3 and 4 from the “Block” experiment (right panel) tended to have higher bark thickness when compare to the other groups.


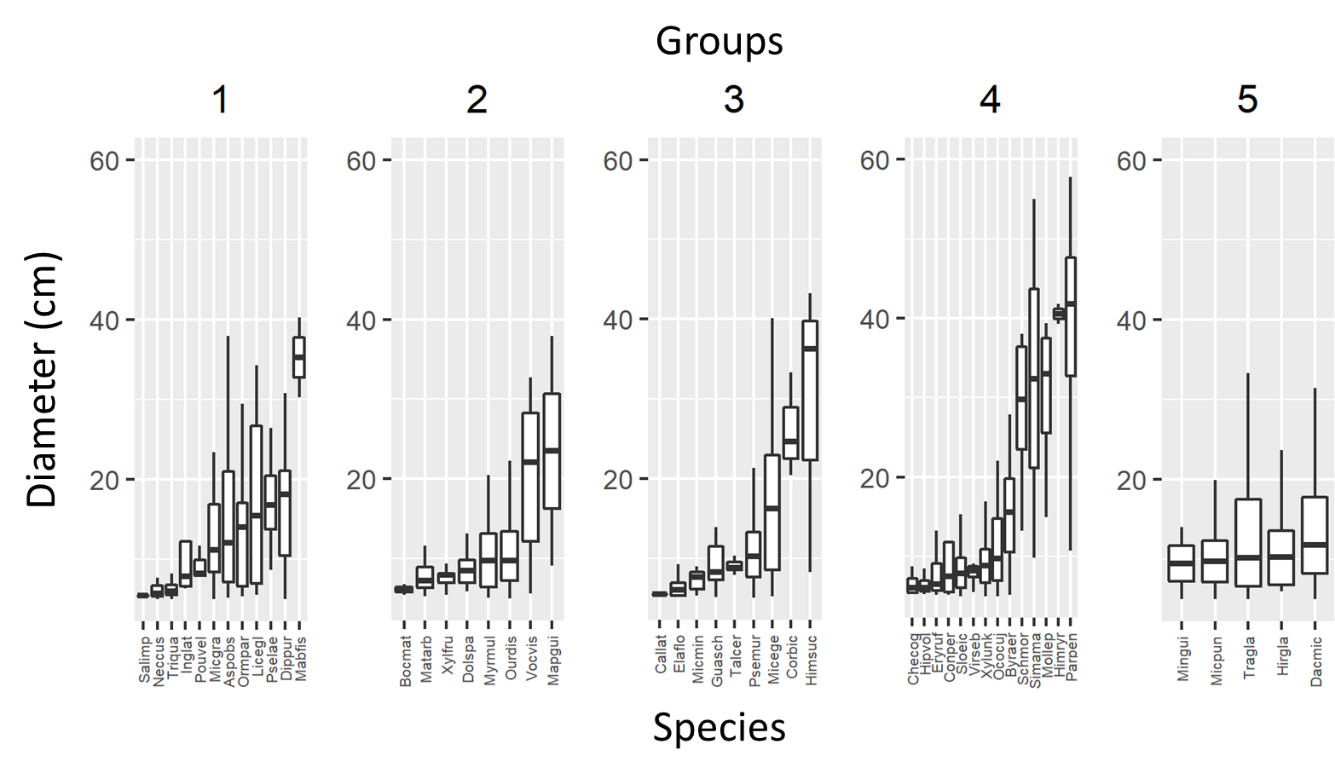


Fig. 2. Diameter distribution for the characteristic species for each group in the “Block” experiment. Notice that all the characteristic species of group 5 have relatively smaller diameters whereas the other groups are comprised of characteristic species with small and large diameters.
