# Supplementary material for: The Latent Dirichlet Allocation model with covariates (LDAcov): A case study on the effect of fire on species composition in Amazonian forests
Source: Ecol Evol. 2021 May 5;11(12):7970–9. doi: 10.1002/ece3.7626 (PMC8216892; doi:10.1002/ece3.7626)
Supplement: Supplementary file 5 — Appendix S5 [file ECE3-11-7970-s001.docx]

**Appendix 5. Interpretability problems with multinomial logistic regression**

The Regions of Common Profile model (RCP; Foster *et al.*, 2017, Lyons *et al.*, 2017) relies on a multinomial logistic regression model to relate the covariates at each site $\boldsymbol{x}_{\boldsymbol{i}}$ with the probability of that site belonging to each cluster k $\pi_{ik}$. This model assumes that $\pi_{ik}$ is given by

$\pi_{ik}=\frac{\exp\left( x_{i}^{T}\beta_{k} \right)}{1+\sum_{c=1}^{K-1} \exp\left( x_{i}^{T}\beta_{c} \right)}$ if $1\leq k\leq K-1$, and

$\pi_{ik}=\frac{1}{1+\sum_{c=1}^{K-1} \exp\left( x_{i}^{T}\beta_{c} \right)}$ if k=K,

where $\boldsymbol{x}_{\boldsymbol{i}}$ be the design vector for sampling unit i, containing a leading 1 for the intercept and the covariates specific to that sampling unit.

The Structural Topic Model (STM; Roberts *et al.*, 2016) relies on a similar regression approach but it allows for additional uncertainty by assuming a logistic normal distribution. More specifically, for a model with K topics, the logistic normal is given by

$$\boldsymbol{\eta}_{\boldsymbol{i}}\sim N_{k-1}\left( \boldsymbol{\mu}_{\boldsymbol{i}}\boldsymbol{,}\boldsymbol{\Sigma} \right)$$

$$\pi_{ik}=\frac{\exp\left( \eta_{ik} \right)}{1+\sum_{c=1}^{K-1} \exp\left( \eta_{ic} \right)}$$

where $\boldsymbol{\mu}_{\boldsymbol{i}}\boldsymbol{=}\boldsymbol{x}_{\boldsymbol{i}}^{\boldsymbol{T}}\mathbf{B}$. In this expression, $\mathbf{B=[}\boldsymbol{\beta}_{\mathbf{1}}\boldsymbol{|\ldots}\left| \boldsymbol{\beta}_{\boldsymbol{K-1}} \right]$ is a $(K-1)\times P$ matrix containing the regression coefficients for all groups 1,…,K-1.

To illustrate the difficulty associated with interpreting the regression coefficients $\boldsymbol{\beta}_{\mathbf{k}}$, we assume that there are only three groups and a single covariate (drawn from a uniform distribution between -2.5 and 2.5). We will also assume that groups 1 and 2 have the same intercept, equal to 0, but that group 1 has a slope of 1 while group 2 has a slope of 2 (i.e., $\mathbf{B=}\left[ \begin{matrix} 0 & 0 \\ 1 & 2 \end{matrix} \right]$). Finally, we also assume that the variances are small and that there is no correlation between groups (i.e., $\boldsymbol{\Sigma=}0.01\mathbf{I}$).

A naïve interpretation of the slope parameter for group 1 would suggest that the prevalence of this group is positively associated with the covariate given that this slope is equal to 1. However, depiction of simulated data clearly shows that this is not the case. Despite a positive slope, we find a relationship between the covariate and the prevalence of group 1 that originally is positive but subsequently becomes negative, both for RCP and STM (Fig. 1).

zz
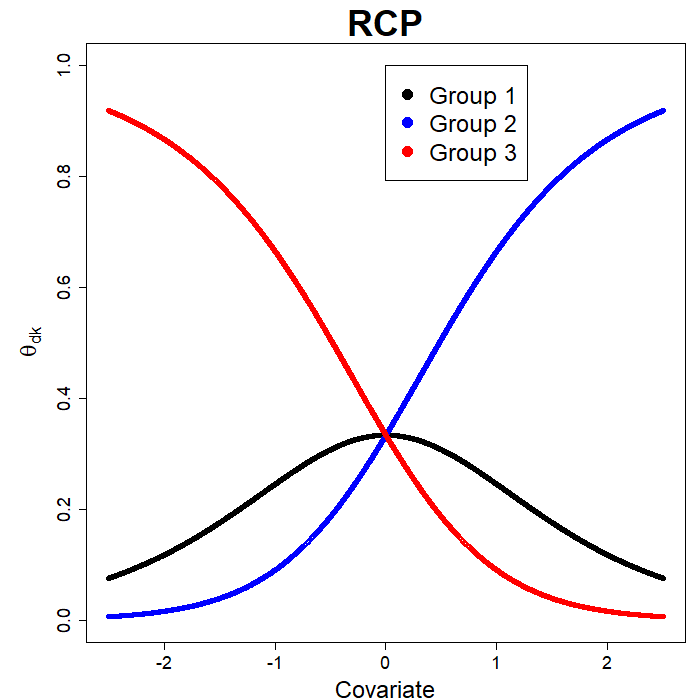

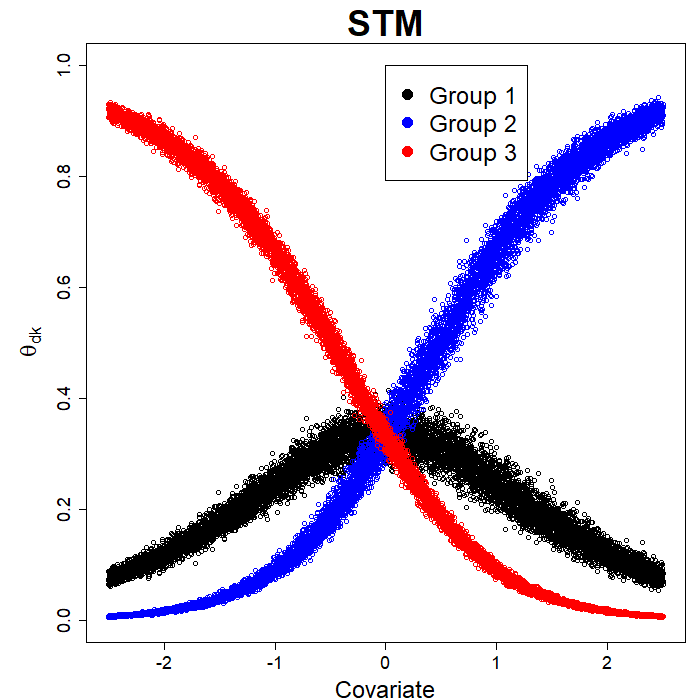


Fig. 1. Relationship between the prevalence of each group (shown with different colors) as a function of the covariate, based on the RCP and STM assumptions (left and right panels, respectively).

**References**

Foster SD, Hill NA, Lyons M (2017) Ecological grouping of survey sites when sampling artefacts are present. Royal Statistical Society: Applied Statistics Series C.

Lyons MB, Foster SD, Keith DA (2017) Simultaneous vegetation classification and mapping at large spatial scales. Journal of Biogeography, 1-12.

Roberts ME, Stewart BM, Airoldi EM (2016) A model of text for experimentation in the social sciences. Journal of the American Statistical Association*,* **111**, 988-1003.
